# Defect Engineering with Rational Dopants Modulation for High-Temperature Energy Harvesting in Lead-Free Piezoceramics

**DOI:** 10.1007/s40820-024-01556-5

**Published:** 2024-11-04

**Authors:** Kaibiao Xi, Jianzhe Guo, Mupeng Zheng, Mankang Zhu, Yudong Hou

**Affiliations:** Key Laboratory of Advanced Functional Materials, Ministry of Education, College of Materials Science and Engineering, Beijing University of Technology, Beijing, 100124 People’s Republic of China

**Keywords:** Lead-free piezoceramic, Defect engineering, Dopants modulation, High-temperature, Piezoelectric energy harvester

## Abstract

High temperature piezoelectric energy harvester (HT-PEH) is an important solution to replace chemical battery to achieve independent power supply of HT wireless sensors. However, simultaneously excellent performances, including high figure of merit (FOM), insulation resistivity (*ρ*) and depolarization temperature (*T*_d_) are indispensable but hard to achieve in lead-free piezoceramics, especially operating at 250 °C has not been reported before. Herein, well-balanced performances are achieved in BiFeO_3_–BaTiO_3_ ceramics via innovative defect engineering with respect to delicate manganese doping. Due to the synergistic effect of enhancing electrostrictive coefficient by polarization configuration optimization, regulating iron ion oxidation state by high valence manganese ion and stabilizing domain orientation by defect dipole, comprehensive excellent electrical performances (*T*_d_ = 340 °C, *ρ*_250 °C_ > 10^7^ Ω cm and FOM_250 °C_ = 4905 × 10^–15^ m^2^ N^−1^) are realized at the solid solubility limit of manganese ions. The HT-PEHs assembled using the rationally designed piezoceramic can allow for fast charging of commercial electrolytic capacitor at 250 °C with high energy conversion efficiency (*η* = 11.43%). These characteristics demonstrate that defect engineering tailored BF-BT can satisfy high-end HT-PEHs requirements, paving a new way in developing self-powered wireless sensors working in HT environments.

**Supplementary Information:**

The online version contains supplementary material available at 10.1007/s40820-024-01556-5.

## Highlights

- The solution limit of manganese ion in BiFeO_3_–BaTiO_3_ (BF–BT) was determined by combining multiple advanced characterization methods.
- The defect engineering associated with fine doping can realize the co-modulation of polarization configuration, iron oxidation state and domain orientation.
- The BF–BT–0.2Mn piezoelectric energy harvester shows excellent power generation capacity at 250 °C, which is an important breakthrough for lead-free piezoelectric devices.

**Supplementary Information:**

The online version contains supplementary material available at 10.1007/s40820-024-01556-5.

## Graphical abstract

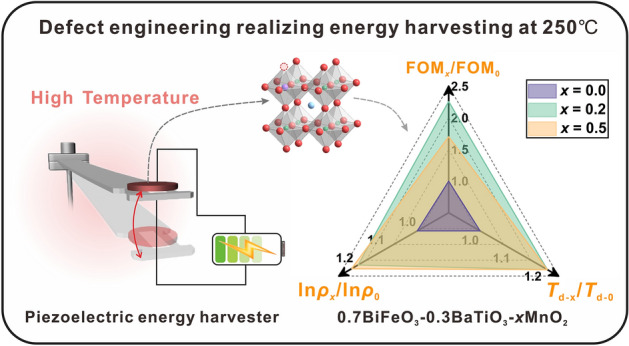

## Introduction

**Fig. 1:**
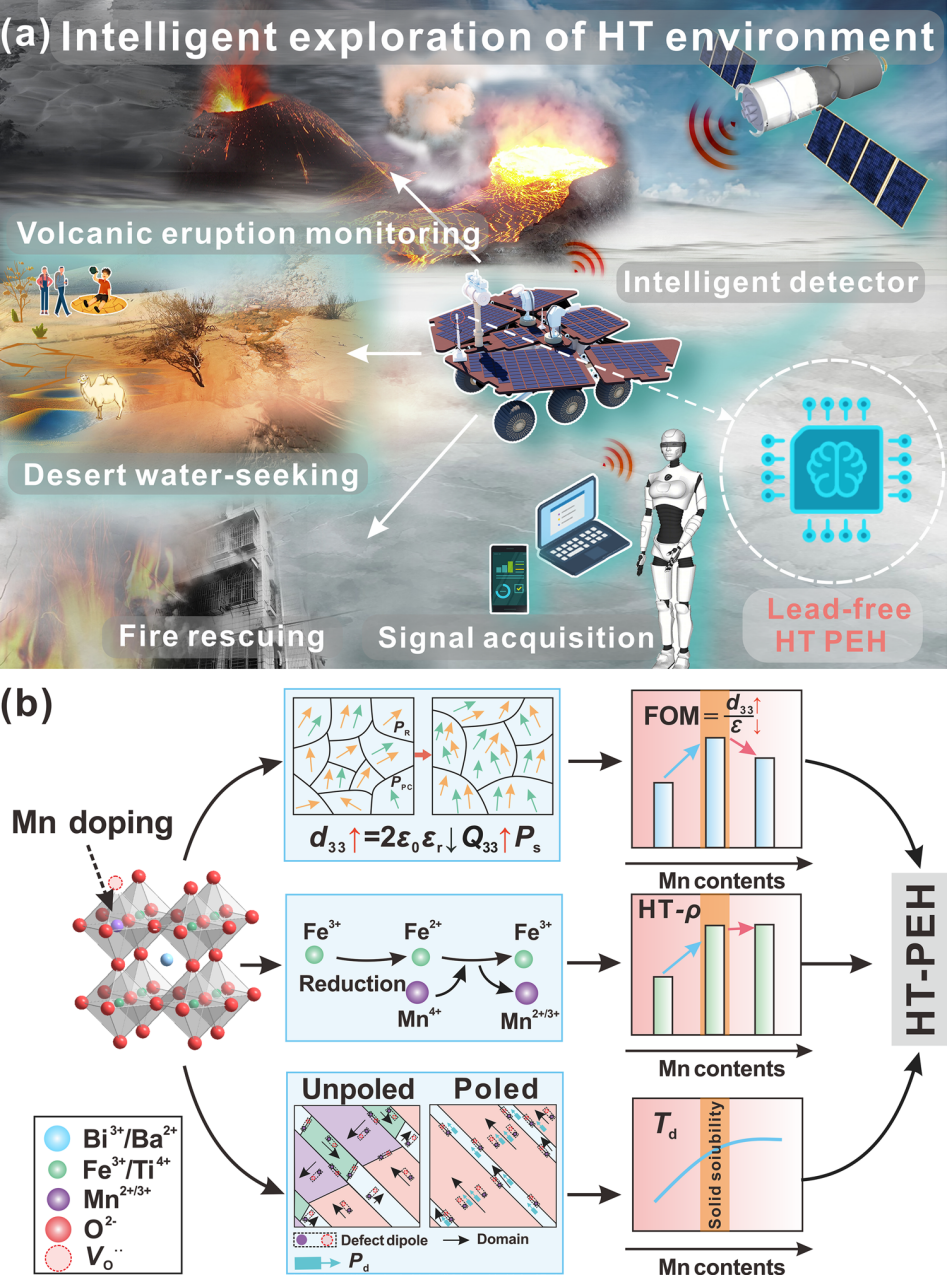
**a** Intelligent monitoring scene of HT-environment; **b** Schematic diagram of defect engineering used to build a HT-PEH with manganese doped BF-BT ceramic system

The realization of lead-free piezoelectric devices is urgently needed for environmental sustainability [1, 2]. Although new types of lead-free piezoceramics have emerged in the past few decades, and there have been some cases of application in conventional piezoelectric devices such as commercial lead-free transducers [3–5], but in the field of high-end piezoelectric devices represented by high temperature piezoelectric energy harvester (HT-PEH), lead-free piezoceramics are still difficult to find. In the intelligent exploration of HT environment fields, such as volcanic eruption monitoring, desert water-seeking and fire rescuing, high temperature wireless sensors are widely used in the intelligent detector, which are eager to use lead-free HT-PEH to achieve self-powered supply (Fig. 1a).

HT-PEH is an important solution for realizing long-term self-power of high temperature wireless sensors, because it not only converts the low-frequency vibration energy generated by the machine itself into electricity, but also is safer in high temperature environments than existing chemical battery power models [6, 7]. Unfortunately, the HT-PEH material that can achieve stable power generation above 250 °C is still dominated by PbTiO_3_–BiScO_3_ system (PT-BS) [8], which not only brings high piezoelectric properties due to the morphotropic phase boundary (MPB) composition like Pb(Zr,Ti)O_3_ (PZT), but more importantly, compared with PZT counterpart, its Curie temperature (*T*_C_) is increased by more than 100 °C, which brings better anti-depolarization characteristics [9]. In the lead-free piezoceramics family, BiFeO_3_–BaTiO_3_ ceramics (BF–BT) with high *T*_C_ and high piezoelectric properties stand out and have attracted great attention in recent years [10–12]. However, there are significant challenges to applying HT-PEH, including the need to further increase the thermal depolarization temperature (*T*_d_) and figure of merit (FOM) associated with the power generation of PEH, while ensuring high temperature insulation to avoid device failure due to leakage [13]. In this work, these concerns have been addressed by innovative defect engineering [14] strategy with respect to delicate manganese doping (Fig. 1b).

Manganese doping is widely used in the modification of lead-based piezoceramics represented by PZT, which plays acceptor role to improve the mechanical quality factor (*Q*_m_), and is accompanied by the attenuation of piezoelectric charge constant (*d*_33_) and relative dielectric constant (*ε*_r_) [15]. Although several studies have been reported on manganese-doped BF–BT [16, 17], clear defect engineering for HT-PEH applications is still lacking. According to the piezoelectric theory, the generating power is directly related to the piezoelectric charge constant *d*_33_ and the piezoelectric voltage constant *g*_33_, and the product of the two (*d*_33_·g_33_) is defined as the FOM [18]. Considering that *g*_33_ = *d*_33_/*ε*_0_·*ε*_r_, the FOM associated with the power generation of PEH can be expressed in another form:

1 $$ {\text{FOM}}{\kern 1pt} = \frac{{d^{2} }}{{\varepsilon_{0} \varepsilon_{r} }} $$

where *ε*_0_ is the vacuum dielectric constant (8.854 × 10^−12^ F m^−1^).

Generally, the hardening effect of manganese doping on piezoelectric properties will cause the decrease of *d*_33_ and *ε*_r_ at the same time, which is unfavorable to the improvement of FOM [19]. Therefore, one of the key problems to be solved in this study is how to decouple the synergistic change between *ε*_r_ and *d*_33_, that is, how to increase *d*_33_ while reducing *ε*_r_. As we known, in perovskite ferroelectrics, *d*_33_ can be expressed as a thermodynamic calculation-based formula [20]:

2 $$ d_{33} = {\kern 1pt} {\kern 1pt} 2\varepsilon_{0} \varepsilon_{{\text{r}}} P_{{\text{s}}} Q_{33} $$

where *P*_s_ is the spontaneous polarization, *Q*_33_ is the electrostrictive coefficient, which is an intrinsic parameter determined by the lattice structure of the material.

Take into account of the thermodynamic constraint relationship between the above electrical parameters, we speculate that the appropriate introduction of manganese ions into the BF–BT perovskite structure can optimize the polarization configuration with increasing B–O bond length, which is expected to enhance *P*_s_ and *Q*_33_ (a ferroelectric material with a larger relative displacement of the B–O bond length would possess a higher *Q*_33_ [21, 22]), helping compensate for the negative effects of decreasing *ε*_r_ [23], thus making it possible to obtain high *d*_33_ and large FOM (Fig. 1b). Another problem that seriously affects the application of BF-BT at high temperature is the fluctuation of valence of Fe ions in ceramics prepared by conventional sintering, especially the high content of Fe^2+^ ions lead to poor insulation performance of the material [24]. In response to this issue, the introduced MnO_2_ is expected to play a strong oxidant role, promoting the transformation of Fe^2+^ ions to Fe^3+^ ions, so as to stabilize the high-temperature insulation resistivity (Fig. 1b) [17]. Finally, as the acceptor center, low-valence Mn^2+^/Mn^3+^ ions can combine with adjacent oxygen vacancies to form defect dipoles [25, 26]. For artificially polarized piezoceramics, the internal electric field (*P*_d_) induced by these defect dipoles is expected to stabilize the domain orientation, thus enhancing the resistance to thermal depolarization (Fig. 1b). In order to verify the feasibility of the above defect engineering strategy, we selected MnO_2_ as the dopant to modify the BF-BT system, focusing mainly on the changes in the phase composition, microstructure and electrical properties of the material before and after the solid solubility limit of manganese ions. In addition to the in-depth analysis of the underlying defect mechanism, it is exciting to see that the cantilever beam type PEH assembled with optimized Mn modified BF-BT materials exhibits excellent power generation capacity at 250 °C, which shed new light on the development of high performance lead-free piezoceramics meeting the demands of high-end piezoelectric devices.

## Experimental

The composition designed in this work is 0.70BiFeO_3_-0.30BaTiO_3_-*x* mol% MnO_2_ (referred to as BF-BT-*x*Mn; *x* = 0.0, 0.1, 0.2, 0.3, 0.4, 0.5), which were fabricated by using the conventional solid-state reaction method. The starting raw materials of Bi_2_O_3_ (99%), Fe_2_O_3_ (99%), BaCO_3_ (99%), TiO_2_ (98%) and MnO_2_ (85%) were mixed by the planetary ball milling in ethanol at 400 r min^−1^ for 12 h, using ZrO_2_ balls as milling media. To compensate for the volatilization of Bi_2_O_3_ during sintering, 2 mol% excess of Bi_2_O_3_ was added to the stoichiometric composition. The mixture was dried and calcined at 800 °C for 3 h. Subsequently, the calcined powder was ball milled again for 12 h and then mixed with 5 wt% polyvinyl alcohol (PVA), before being pressed into pellets of 11.5 mm in diameter and 1.0 mm in thickness under 100 MPa. After burning off the PVA binder at 560 °C for 3 h, the green pellets were sintered in an alumina crucible at a temperature of 980–1000 °C for 3 h, buried in sacrificial powder with the same composition. The sintering temperatures were slightly adjusted for each composition for optimization.

The bulk density of the ceramic was determined using the Archimedes drainage method. The microscopic morphology and elemental distribution of plane sections of the sample after acid etching were observed by scanning electron microscopy (SEM; S4800, Hitachi, Tokyo, Japan) equipped with energy-dispersive X-ray spectroscopy (EDS). The acid etching solution is composed of aqueous HCl acid (with a mass concentration of about 37%) and aqueous HF acid (with a mass concentration of 40%), which were mixed according to the volume ratio of 1:1. The grain size was measured and counted using Nano Measurer software. The phase composition was studied by X-ray diffractometer (XRD, Bruker D8 Advance, Karlsruhe, Germany) in the *θ*–2*θ* configuration using Cu Kα radiation. Rietveld refinements were performed using TOtal PAttern Solution (TOPAS) software. The symmetry of the microscopic local structure of the ceramic was investigated using Raman Scattering Spectroscopy (HR800, Japan) with a wavelength range of 100 ~ 1000 cm^−1^. The valence state proportions of Mn and Fe elements in the sample were determined by X-ray photoelectron spectroscopy (XPS, ESCALAB 250, Thermo-VG Scientific, America). The domain configurations and selected area electron diffraction (SAED) patterns were investigated using transmission electron microscope (TEM; Tecnai F30, FEI, Hillsboro, OR). Besides, the domain configuration was also observed using the piezoresponse force microscopy (PFM) carried out on a polished ceramic sample by an atomic force microscope (Bruker Dimension Icon, US) with an SCM-PIT-V2 conductive probe (Bruker, US).

For electrical measurements, both surfaces of the polished ceramics were painted with silver paste and then fired at 550 °C for 20 min to form electrodes. All samples were poled at 90 °C in a silicone oil bath with an applied DC electric field of 4–5 kV mm^−1^ for 20 min. After aging for 24 h, the room temperature *d*_33_ values were measured by using a quasi-static Berlincourt meter (ZJ-6A, Institute of Acoustics, Chinese Academy of Sciences, China) at 100 Hz. To accurately evaluate the real-time temperature dependence of *d*_33_, the in situ* d*_33_ was measured on piezoceramics at elevated temperatures with a custom-designed wide-temperature-range *d*_33_ test system [7]. Variable temperature dielectric properties were obtained using a multifrequency inductance–capacitance–resistance (LCR) analyzer (E4980A, Agilent Technologies, Santa Clara, CA, USA) with an automatic temperature controller. The mechanical quality factor (*Q*_m_) and electromechanical coupling factor (*k*_p_) were determined by a precision impedance analyzer (4294A; Agilent Technologies, Santa Clara, CA, USA) through the resonance anti-resonance method based on IEEE standards. The ferroelectric hysteresis loops (*P*–*E*) and bipolar strain curves (*S*–*E*) were measured using a ferroelectric instrument (CPE1801, PolyK Technologies, LLC) equipped with a photonic sensor (MTI-2000, MTI, Albany, NY) operating at 1 Hz. The DC resistivity was tested by an electrometer (Keithley 6517B) with an automatic temperature controller. A broadband impedance spectrometer (Concept 40, Novocontrol Technologies, Montabaur, Germany) was used to measure the temperature-dependent insulation resistivity.

In order to directly evaluate the energy harvesting characteristic of the samples at different temperature, cantilever beam type PEHs test system has been designed. The precision processed (*ϕ*10.15 mm × 0.49 mm) and polarized sample with lead wire was fixed on the gradient cantilever beam (350 mm × 12 mm × 2 mm) by screw. One end of the cantilever beam (free end) is placed in a heating furnace (KSL-1100X-S, Hefei Kejing Material Technology Co., Ltd, China), and the other end (fixed end) is fixed on the shaking table (Model K2007E01; The ModalShop Inc, Cincinnati, USA). The vibration acceleration of PEHs was measured by a charge amplifier (MI2004, Econ Technologies Co., Ltd, China) and a piezoelectric accelerometer (Model 3211A, Dytran Instrument Inc, California, USA). The output voltage of PEHs was measured using a digital oscilloscope (MDO3024; Tektronix, USA). The output current of PEHs was obtained by a low noise current preamplifier (SR570, Stanford, USA) [7].

## Results and Discussion

### Identification of Solid Solution Limitation of Mn Addition

**Fig. 2:**
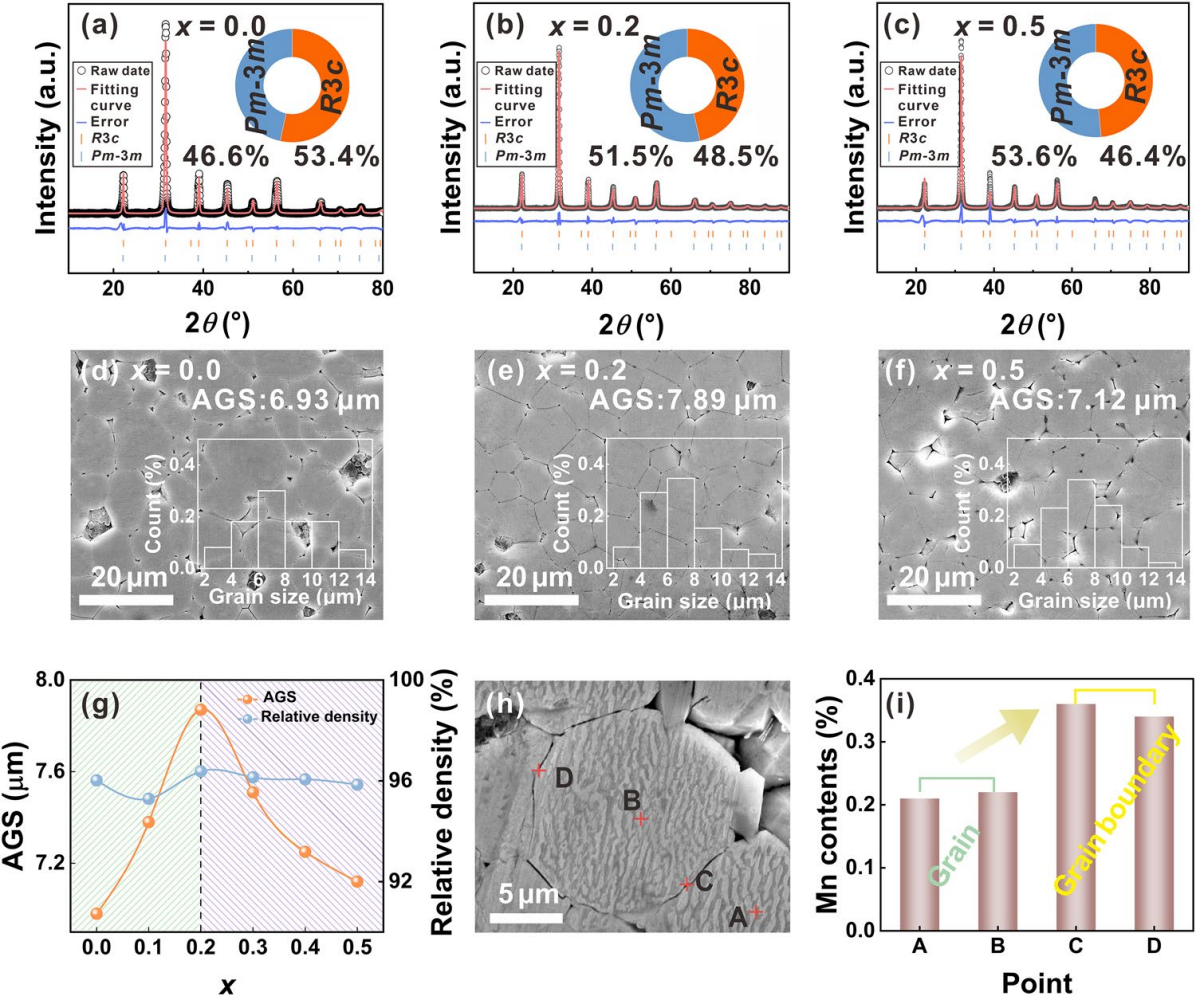
**a**–**c** Rietveld refinement results of XRD for *x* = 0.0, 0.2, and 0.5 samples; **d**–**f** SEM images of the polished and acid etched cross section of the *x* = 0.0, 0.2, and 0.5 samples, and the insets show the corresponding grain size distribution; **g** AGS and the relative density of the BF-BT-*x*Mn ceramics; **h** SEM photograph marked with the location of selected test points; **i** EDS point scan results of *x* = 0.5 sample

It is known that the lattice structure of MnO_2_ and BF–BT is very different, so there is a certain solid solution limit of Mn ion in the perovskite matrix [16]. Some studies have revealed that when the content of Mn ion is below or above the solid solution limit, it has different effects on the lattice distortion and grain size of perovskite ferroelectrics, and then has different effects on the electrical properties [26]. In this work, firstly, the effect of Mn ion on the phases structure of BF-BT ceramics was analyzed. Figure S1 displays the room-temperature XRD patterns of the BF-BT-*x*Mn ceramic, along with the standard diffraction peaks of BT with *PC* symmetry (*Pm*-3*m*, PDF#75-0461) and BF with *R* symmetry (*R*3*c*, PDF#71-2494) [27]. All samples present mainly a perovskite structure without the appearance of impurity phase. However, in the doping system below *x* = 0.2, the left shift of the characteristic diffraction peak near 31°–32° can be clearly observed as the Mn content increases (Fig. S1), which indicates that appropriate addition of Mn ions causes the lattice expansion. It is well-known that BF-BT matrix belongs to ABO_3_ type perovskite oxide, in which Bi^3+^ ions (CN = 12, 1.45 Å) and Ba^2+^ ions (CN = 12, 1.35 Å) occupy the A position, and Fe^3+^ ions (CN = 6, 0.645 Å) and Ti^4+^ ions (CN = 6, 0.605 Å) occupy the B position. For MnO_2_ dopants, the intrinsic valency of Mn ion is + 4, but under the effect of high temperature reduction reaction, Mn^3+^ (CN = 6, 0.645 Å) and Mn^2+^ ions (CN = 6, 0.83 Å) coexist in the sintered body [28]. The determination of the accurate valency state of Mn ion and the relevant reduction reaction mechanism will be given later. In this case, it can be determined that the substitution of large-size doped Mn^2+^/Mn^3+^ ions on small-size B-site ions in perovskite matrix is the main reason for lattice expansion. When the Mn doping amount is higher than *x* = 0.2, the characteristic diffraction peak turns to shift to the right side, preliminarily inferring that the doping amount of *x* = 0.2 is close to the solid solution limit. To accurately determine the lattice parameters and phase content changes of BF-BT-*x*Mn ceramics, samples with three different compositions (*x* = 0.0, 0.2, and 0.5) were selected for XRD refinement analysis. The refinement results are presented in Fig. 2a–c and Table 1. It is observed that all three samples exhibit a coexistence of *R* and *PC* phases, suggesting their location in the MPB region. Moreover, as the Mn content increases, a gradual transition from the *R*3*c* to *Pm*-3*m* phase occurs. In particular, sample located at the *x* = 0.2 has close two-phase content (*R*3*c*, 48.5%; *Pm*-3*m*, 51.5%), which is speculated to contribute to improving piezoelectric properties, since thermodynamic studies have revealed that close two-phase content in ferroelectric solid solutions enables Landau free energy curve flattening associated with polarization [29]. Moreover, to explore the effect of Mn ion content on grain size, the SEM images of BF-BT-*x*Mn samples are shown in Figs. S2a–c and 2d–f. The grains in all samples are well grown and the grain boundary angle is about 120°, and all samples present dense microstructure with high density (Fig. S2d) and relative density higher than 95% (Fig. 2g). At the same time, with the increase in Mn content, the average grain size (AGS) of the sample increases first and then decreases, with an inflection point at *x* = 0.2 (Fig. 2g). Below *x* = 0.2, Mn ions enter the perovskite matrix and increase the amount of lattice defects, thus promoting grain growth due to enhanced material transport. The fast grain growth of Mn doped BF-BT can be mainly attributed to the formation of oxygen vacancies that promotes the lattice diffusion [30]. In contrast, above *x* = 0.2, excessive Mn ions are enriched in grain boundaries, and these dopants nail grain boundary migration, resulting in grain size reduction [31]. Furthermore, take *x* = 0.5 sample as example, EDS analysis of focused Mn content was performed at four points where A and B are located inside the grain, and C and D are located at the grain boundary (Fig. 2h). The EDS point scan results show that the content of Mn ion in the grain is about 0.2 mol% (Fig. 2i), agreeing well with the inflection point of AGS change indicated in Fig. 2g. Therefore, based on the above phase/microstructure evolution and elemental analysis results, it is inferred that the solid solution limit of Mn ion in BF-BT matrix is around *x* = 0.2.

**Table 1:** Refined lattice parameters and phase content of BF-BT-*x*Mn ceramics

*X*	Lattice parameters			Phase content (%)		*R*_wp_ (%)
	*R*3*c*		*Pm-*3* m*	*R*3*c*	*Pm-*3* m*	
	*a* (Å)	*c* (Å)	*a* = *b* = *c* (Å)			
0.0	5.6388	13.8873	4.0109	53.406	46.594	4.64
0.2	5.6631	13.8946	4.0144	48.503	51.497	3.77
0.5	5.6429	13.8370	4.0119	46.388	53.612	6.68

### Boosting FOM by Optimized Polarization Configuration

**Fig. 3:**
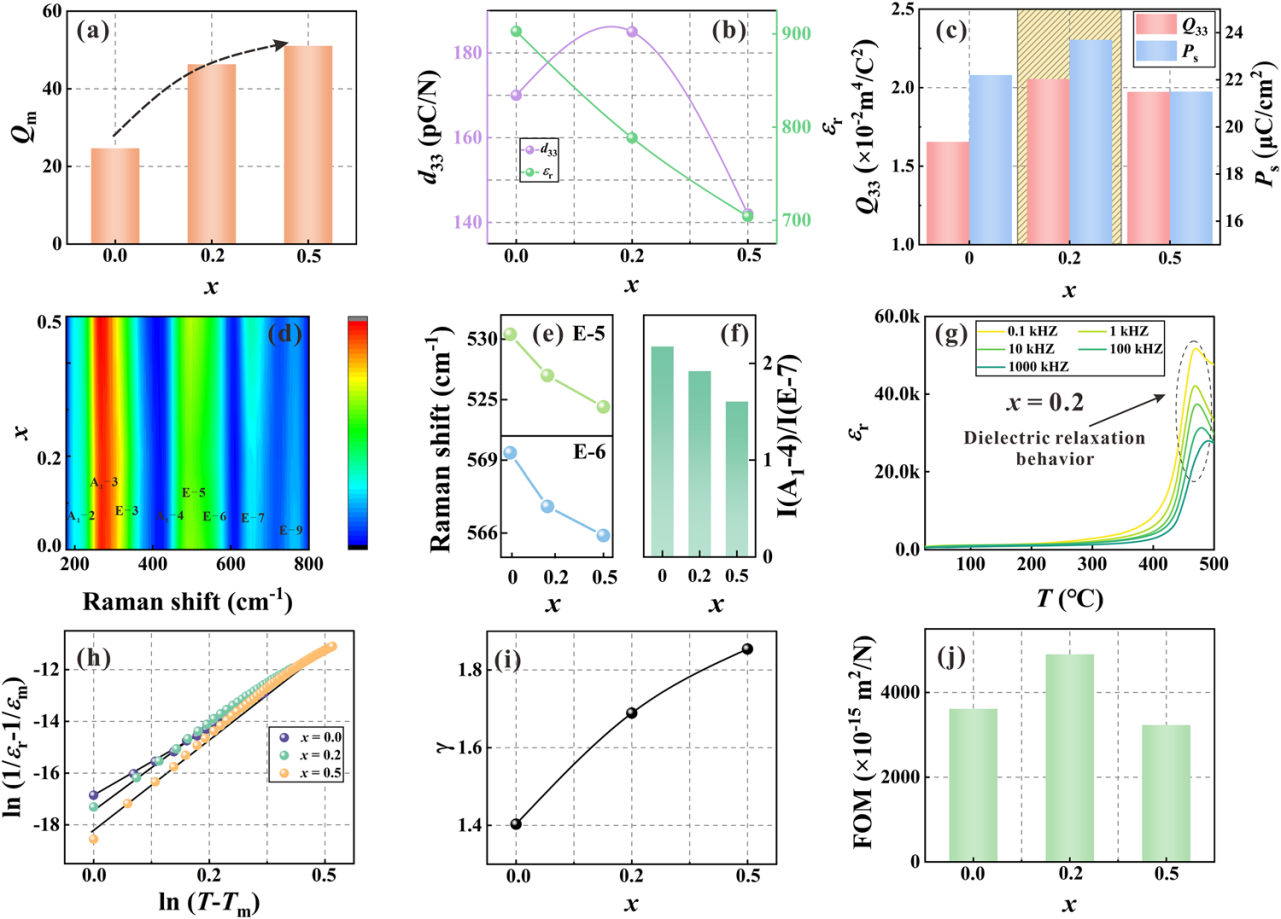
**a** *Q*_m_ value of the* x* = 0.0, 0.2 and 0.5 samples; **b** *d*_33_ and *ε*_r_ of the three ceramics; **c** *Q*_33_ and *P*_s_ of the three ceramics; **d** Raman spectrum with Gaussian fitting curves; **e** Raman shift of E-5 and E-6 vibrational mode peaks; **f** Intensity ratio of I(A_1_-4)/I(E-7) of the three ceramics; **g** *ε*_r_-*T* curves of *x* = 0.2 sample measured at different frequency; **h** The fitting results based on modified Curie–Weiss law; **i** *γ* value of the three ceramics; **j** FOM value of the three ceramics

As we know, piezoceramics suitable for PEH should have a high *d*_33_, because it is closely related to the power generation. However, Mn doping mainly plays a hardening role in perovskite system, which is related to the inhibition of domain wall motion by defect dipoles [32], and BF-BT is no exception. As can be seen from Fig. 3a, Mn doping causes a continuous increase in *Q*_m_ value, and accompanied by the attenuation of *ε*_r_ (Fig. 3b), which is a typical manifestation of hardening effect. Otherwise, the *d*_33_ shows a trend where it increases initially and subsequently decreases, reaching a maximum value at *x* = 0.2 (Fig. 3b). According to formula (2), the main way to enhance *d*_33_ needs to start from the internal factor, that is, to achieve high *Q*_33_ and *P*_s_ through crystal structure modulation. Further, *P*_s_ and *Q*_33_ of different samples can be extracted from *P*-*E* loops and *S*-*P* curves (Fig. S3a, b), and the results are shown in Fig. 3c. Excitingly, the extreme values of *Q*_33_ and *P*_s_ are obtained when the Mn doping amount is at the solid solution limit (*x* = 0.2). Here, greater attention should be paid to the fact that the *Q*_33_ is an intrinsic effect that is primarily dependent on the inherent crystal structure and the valence bonds [33]. Considering that Raman spectroscopy is more sensitive to the local symmetry, we conducted Raman tests on the three samples with *x* = 0.0, 0.2, and 0.5, and the results are present in Fig. 3d. As known, BiFeO_3_ with *R*3*c* has 13 Raman active vibration modes Γ_R_ (*R*3*c*) = 4A_1_ + 9E, in which A is a non-degenerate state with respect to the main axis, and E is double degenerate state [34]. For the Mn-doped BF-BT system, only eight vibration modes were fitted because some vibration modes were weak or overlapped. The E-4 and E-8 modes disappear after Mn addition, revealing the evolution of local structure, which corresponding to the phase transformation from *R*3*c* to *Pm*-3* m* [16]. Further, Mn addition induced the red shift of E-5 and E-6 vibrational mode peaks as seen in Fig. 3e. The red-shift of E-5 and E-6 modes indicates that the Mn-doping weakens the hybridization between the empty Fe^3+^/Ti^4+^ *d*-orbitals and the adjacent oxygen *p*-orbitals [17], reducing the bond strength of B–O bond with increasing B–O bond length (Table S1) and beneficial to the improvement of *Q*_33_. Figure 3f further shows the peak intensity ratio between the two modes A_1_-4 and E-7. As the Mn content increases, the ratio of I(A_1_-4)/I(E-7) decreases gradually, suggesting that the oxygen octahedral distortion is weakened [34] accompanied with the disturbance of the degree of B-site order, thereby improving the *Q*_33_ value. The decreased *Q*_33_ value in *x* = 0.5 may be related to the fact that the doping content exceeds the solid solution limit, resulting in lattice shrinking (Fig. S1). In addition, it can be seen that Mn doping causes the widening of dielectric temperature spectrum of BF-BT ceramics (Figs. 3g and S4), indicating the enhancement of diffuse behavior. The relaxor diffuse factor (*γ*) can reflect the extent of diffuse behavior, which has been calculated according to the modified Curie–Weiss law [35]:

3 $$ \frac{1}{{\varepsilon_{{\text{r}}} }} - \frac{1}{{\varepsilon_{{\text{m}}} }} = \frac{{(T - T_{{\text{m}}} )^{\gamma } }}{C} $$

where *ε*_m_ is the maximum value of dielectric constant, *ε*_r_ is the dielectric constant at temperature *T*, *T*_m_ is the temperature corresponding to *ε*_m_, and *C* is the Curie constant. The value of *γ* is between 1 and 2, with the former representing normal ferroelectrics and the latter representing a relaxor ferroelectric with a complete diffuse phase transition. The fitting results of the three samples are displayed in Fig. 3h and the deduced *γ* of all samples are showed in Fig. 3i. It can be found that the *γ* increases from 1.40 for *x* = 0 to 1.86 for *x* = 0.50, proving the Mn addition enhances the relaxation behavior of the systems. There is a lower polarization reversal potential barrier in the relaxation state [36], allowing easier orientation of *P*_s_ to be achieved under application of the external *E*-field. So, it is speculated that the excellent quality is mainly indebted to optimized polarization configuration arising from close two-phase content coupled with relaxation state, which promote the enhancement of *d*_33_ (Fig. 3b). The non-synergistic change of *d*_33_ and *ε*_r_ resulted in the improvement of FOM (Fig. 3j), which was expected by the previous strategy (Fig. 1b). Moreover, the *k*_p_ of the *x* = 0.2 sample also displayed the maximum value (Fig. S5), showing excellent electromechanical conversion ability, which has the potential to be applied to the PEHs.

### Enhanced HT-*ρ* by Weaking Leakage Conductance Loss

**Fig. 4:**
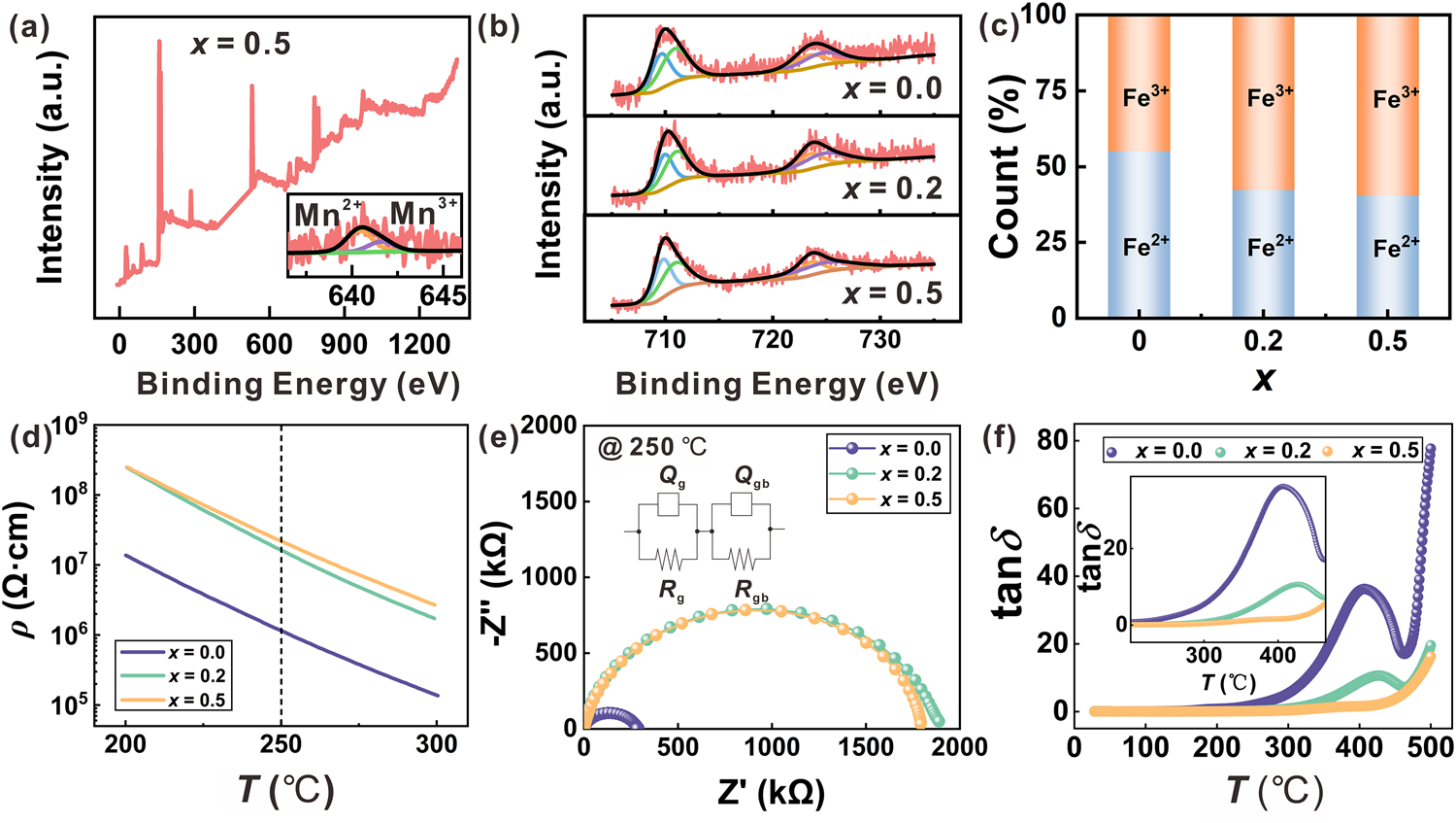
XPS spectra of **a** Mn 2*p* and **b** Fe 2*p*; **c** Fe^2+^/Fe^3+^ relative content with *x* = 0.0, 0.2, and 0.5 samples; **d** Temperature dependent DC resistivity; **e** Cole–Cole plots of the three samples; **f** tan*δ*-*T* curves of BF-BT-*x*Mn ceramics measured at 1 kHz, and the insert shows the local data amplified within the range of 200 to 450 °C

For BF-BT ceramics, the leakage loss at high temperature is mainly related to the movement of oxygen vacancy and the change of valence of Fe ion [32]. Here, the excess bismuth oxide was added to reduce the bismuth and oxygen vacancies caused by volatilization of elements at high temperature [24]. Besides, the control of the valence state of Fe ion mainly depends on the Mn doping effect. Previous inferences were based on the mixed valence state of + 2 and + 3 for Mn ion, and the valence state distribution of Mn ion is a key factor in defect engineering design to weaken leakage conductance loss (Fig. 1b). In order to accurately analyze the mechanism of Mn doping, XPS technique was used to measure the valence distribution of Mn ions in BF–BT–*x*Mn samples. Figure 4a displays the complete XPS spectrum of the *x* = 0.5 sample together with the enlarged pattern corresponding to Mn 2*p*. The results demonstrate that in the sintered samples, the valence state of the Mn element is predominantly + 2, with some presence of + 3, confirming previous assumption on Mn as an acceptor dopant. Figure 4b, c exhibits the XPS peak fitting diagram of Fe 2*p* and the deduced proportions of Fe^2+^ and Fe^3+^ content, respectively. Notably, the result illustrates a significant increase in the proportion of Fe^3+^ as the Mn element content increases from *x* = 0.0 to *x* = 0.2, indicating effective suppression of the valence change in Fe. According to thermodynamic analysis, Mn^4+^ ions will spontaneously transform into Mn^3+^ ions at high temperature, as shown in Eq. (4):

4 $$ 4{\text{MnO}}_{2} = 2{\text{Mn}}_{2} {\text{O}}_{3} + {\text{O}}_{2} \uparrow $$

In the doped BF-BT system, some Mn^3+^ ions can act as oxidants to further oxidize Fe^2+^ ions, as shown in the following Eq. (5):

5 $$ {\text{Mn}}^{3 + } + {\text{Fe}}^{2 + } = {\text{Mn}}^{2 + } + {\text{Fe}}^{3 + } $$

As a result, Mn^2+^ became the dominant type of Mn ions while the content of Fe^3+^ ion increased. In addition, it can be seen from Fig. 4d that only samples with Mn doping content reaching the solid solution limit and above can maintain insulation resistance (*ρ*) higher than 10^7^ Ω cm at 250 °C, which can be partly attributed to the stabilizing effect of Mn doping on the valence state of Fe^3+^ ions, as expected by the defect engineering strategy (Fig. 1b). In addition, the conductance activation energy (*E*_a_) of *x* = 0.0, *x* = 0.2, and* x* = 0.5 samples is calculated through the DC resistivity to quantitatively account for the evolution of conductance mechanism (Fig. S6a). The result showed that the *E*_a_ values of the *x* = 0.0, *x* = 0.2, and* x* = 0.5 ceramics at the high-temperature ranges (200 °C ≤ *T* ≤ 300 °C) are 0.97, 0.88, and 0.86 eV (Fig. S6b), respectively, similar to *E*_a_ of the oxygen vacancy, indicating that the high-temperature internal conductance of the ceramics is mainly determined by the oxygen vacancies. Compared to *x* = 0.0 sample, the samples with *x* = 0.2 and *x* = 0.5 have smaller *E*_a_ value, this is because that Mn ion can inhibit the valence change of Fe ion and thus reducing the free oxygen vacancy, thereby weakening the leakage loss at high temperature. Figure 4e further displays the AC impedance test results of BF-BT-*x*Mn ceramics at 250 °C. With the increase of Mn doping, the semi-arc becomes larger, indicating that the bulk resistance increases [17]. The Cole–Cole plots of all specimens could be simulated using an equivalent circuit with two elements corresponding to the grain contribution (*R*_g_) and grain boundary contribution (*R*_gb_), as shown in the inset of Fig. 4e. It can be seen that Mn doping can increase *R*_g_ and *R*_gb_ simultaneously, and both of them maintain high values for *x* = 0.2 samples (Fig. S7). Generally, dielectric loss mainly comes from relaxation loss and leakage loss, and the latter plays a leading role in the high temperature region [37]. Figure 4f displays the variation of dielectric loss (tan*δ*) for BF–BT–*x*Mn ceramics at different temperatures, with the local data amplified within the range of 200–450 °C. It can be found that Mn doping is very effective in reducing tan*δ* at high temperature. Compared with the tan*δ* of pure BF-BT sample which jumps at 150 °C, the jump temperature of Mn doped BF-BT samples is delayed by nearly 100 °C. The tan*δ* is caused by the conversion of part of the electric energy in the dielectric into heat energy, and high dielectric loss will cause device failure due to serious spontaneous heat. In this work, the excellent insulation characteristics induced by Mn doping reduces the tan*δ* and help ensure stable sample power generation of PEH at high temperatures, thereby avoiding device failure due to leakage conductance, which is beneficial for HT-PEH applications.

### Improved ***T***_d_ by Maintaining Domain Orientation with Defect Dipole

**Fig. 5:**
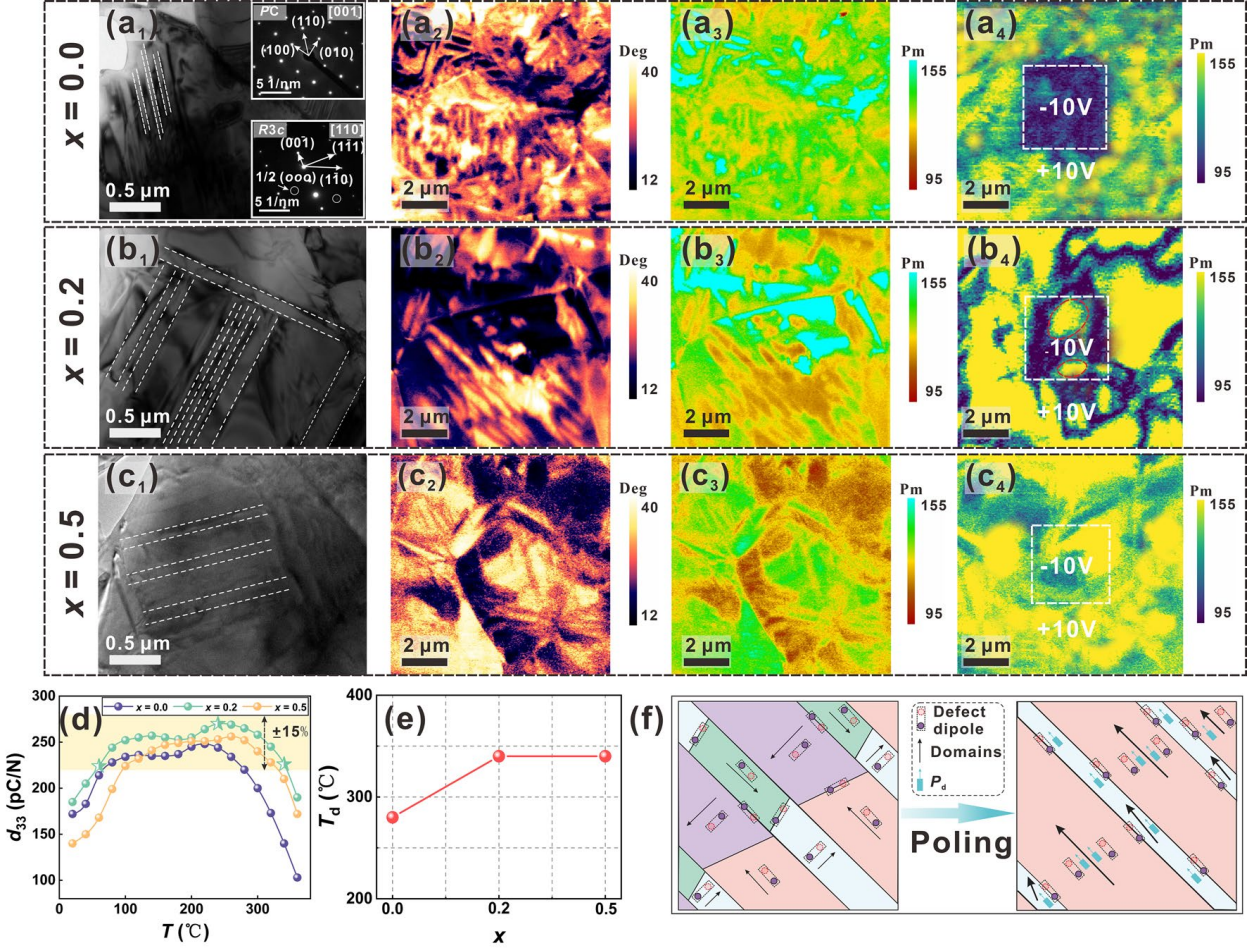
**a**_**1**_–**a**_**3**_ TEM images and PFM images of amplitude and phase for *x* = 0.0 sample, and **a**_**4**_ Amplitude images of “Domain writing” for *x* = 0.0 sample; **b**_**1**_–**b**_**3**_ TEM images and PFM images of amplitude and phase for *x* = 0.2 sample, and **b**_**4**_ Amplitude images of “Domain writing” for *x* = 0.4 sample; **c**_**1**_–**c**_**3**_TEM images and PFM images of amplitude and phase for *x* = 0.5 sample, and **c**_**4**_ Amplitude images of “Domain writing” for *x* = 0.5 sample; **d** In situ measured temperature variable *d*_33_ of BF-BT-*x*Mn ceramics; **e** The composition dependent* T*_d_; and **f** Schematic diagram of proposed mechanism about the improved *T*_d_ via defect dipoles

When considering the application of piezoceramics to HT-PEH, temperature stability of piezoelectric properties is critical, and high *T*_d_ is one of the key guarantees, which is closely related to domain configuration. Here, TEM and PFM are performed to explore the variation of domain configurations with Mn doping. The domain configuration at *x* = 0.0 displays a fragmented morphology with a domain size of 1–2 μm showed in Fig. 5a1–a3. And SAED patterns of the domains are depicted insets of Fig. 5a1, which indicates the coexistence of *R*3*c*-phase and *PC*-phase, consistent with previous XRD and Raman results. As *x* increases to 0.2, the long-range ferroelectric order accompanied by lamellar domains is dominant (Fig. 5b1–b3) and a similar domain configuration also occurs in the *x* = 0.5 sample (Fig. 5c1–c3). Then, a positive voltage and a negative voltage of 10 V were applied to the tip during the scanning of a 4 × 4 μm^2^ area directly, resulting in PFM amplitude images with different colors under + 10 V (outside the white square dashed frame) and − 10 V (inside the white square dashed frame) (Fig. 5a4, b4, c4). The applied voltage of − 10 V is sufficient to create enough domain switching for the ceramic with *x* = 0.0 (Fig. 5a4), but is insufficient for *x* = 0.2 (as marked by the red circle in Fig. 5b4) and *x* = 0.5 samples (Fig. 5c4). The stable large-sized domains may be beneficial to improve the depolarization characteristics, thus optimizing the temperature stability of piezoelectric properties [38]. Figure 5d presents in situ measured temperature variable *d*_33_ of BF-BT-*x*Mn ceramics. Among all compositions, the sample with *x* = 0.2 achieved the highest *d*_33_ value of 270 pC N^−1^ at 250 °C. In this work, the temperature range with 15% deviation from the extreme value of *d*_33_ is considered as the stable temperature region of piezoelectric properties, and the high temperature deviation point is roughly set as the *T*_d_. It was observed that the sample with *x* = 0.2 can sustain a high and stable *d*_33_ value of 270 ± 15% pC N^−1^ over a wide temperature range from 60 to 340 °C, which is very advantageous for high temperature stable current output [39]. In addition, it can be seen from Fig. 5e that appropriate amount of Mn doping contributes to the enhancement of *T*_d_, while there is only slightly reduced for *T*_C_ (Fig. S8). Based on the theory of defect chemistry, Mn acceptor ion and neighbor oxygen vacancy can form defect dipoles. During aging, the defect dipoles will slowly turn to the directions that are parallel (or close) to the spontaneous polarization of the domains, via the short-range diffusion of $V_{O}^{..}$ [25], as shown in Fig. 5f. When upon application of an external electric field, the defect dipoles are aligned along the poling electric field, and the ordered defect dipoles generate a macroscopic internal bias field, which can play an assisting role in promoting domain growth and stabilizing the domain structure oriented in piezoceramics (Fig. 5f), thus enhancing the ability of thermal depolarization resistance [40].

### Mechanism Discussion of Defect Engineering by Mn Doping

The doping effect of Mn on BF-BT ceramics can be described as follows:

6 $$ 2{\text{MnO}}\mathop{\longrightarrow}\limits^{{{\text{Fe}}_{2} {\text{O}}_{3} }}2{\text{Mn}}^{\prime }_{{{\text{Fe}}}} + 2{\text{O}}^{X}_{{\text{O}}} + {\text{V}}_{{\text{O}}}^{ \cdot \cdot } $$

7 $$ {\text{MnO}}\mathop{\longrightarrow}\limits^{{{\text{TiO}}_{2} }}{\text{Mn}}^{\prime\prime}_{{{\text{Ti}}}} + {\text{O}}^{X}_{{\text{O}}} + {\text{V}}_{{\text{O}}}^{ \cdot \cdot } $$

8 $$ {\text{Mn}}_{2} {\text{O}}_{3} \mathop{\longrightarrow}\limits^{{2{\text{TiO}}_{2} }}2{\text{Mn}}^{\prime}_{{{\text{Ti}}}} + 3{\text{O}}^{X}_{{\text{O}}} + {\text{V}}_{{\text{O}}}^{ \cdot \cdot } $$

**Fig. 6:**
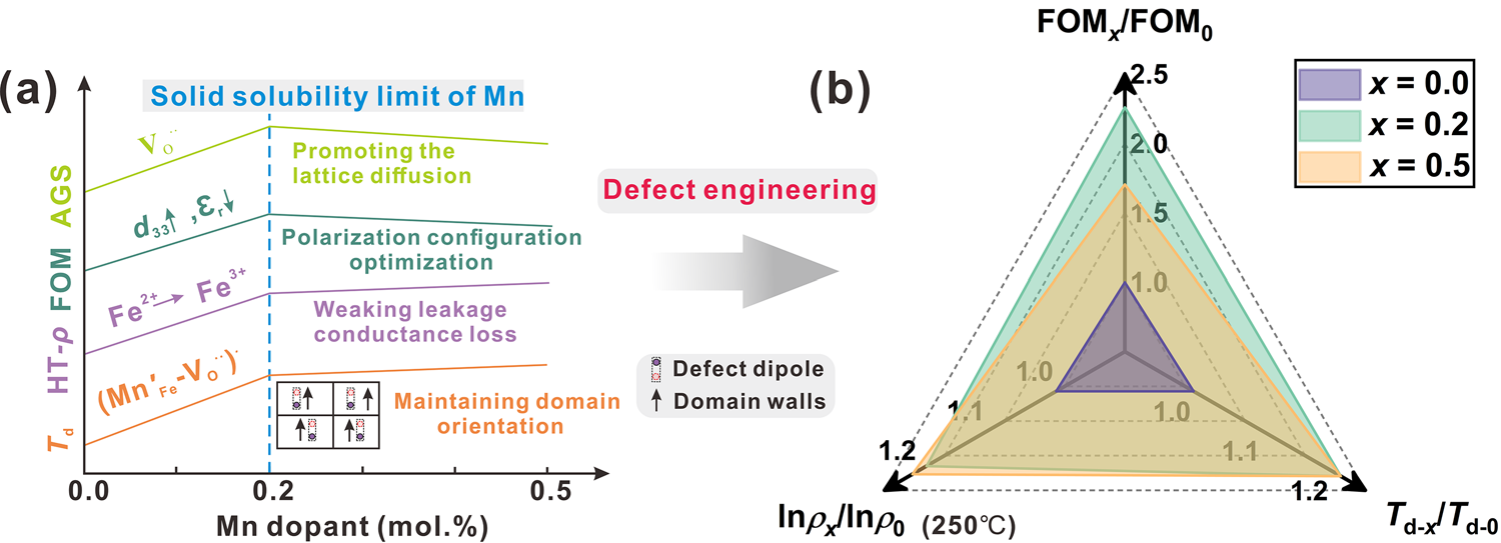
**a** Schematics of efficiency of different mechanisms, AGS, FOM, HT-*ρ* and *T*_d_ as a function of Mn doping amount; **b** Normalized comparison about FOM, ln*ρ* at 250 °C and *T*_d_ of *x* = 0.0, *x* = 0.2, and *x* = 0.5 samples

The formation of ${\text{Mn}}^{\prime }_{{{\text{Fe}}}}$, ${\text{Mn}}^{\prime\prime}_{{{\text{Ti}}}}$ and ${\text{Mn}}^{\prime}_{{{\text{Ti}}}}$ acceptor center is compensated by the formation of $V_{O}^{..}$, and they can form the possible types of defect dipoles, for example, $({\text{Mn}}^{\prime}_{{{\text{Ti}}}} - V_{O}^{..} )^{.}$, $({\text{Mn}}^{\prime}_{{{\text{Fe}}}} - V_{O}^{..} )^{.}$ and $({\text{Mn}}^{\prime\prime}_{{{\text{Ti}}}} - V_{O}^{..} )^{X}$. Here, a simple model is described to illustrate the variation of defect concentration on the AGS, FOM, HT-*ρ* and *T*_d_ of BF-BT samples as a function of the Mn doping amount (Fig. 6). Below the solid solution limit of Mn addition (*x* = 0.2), the formation of $V_{O}^{..}$ promoted the lattice diffusion and enhanced material transport, thereby increasing the AGS and obtaining the maximum value at *x* = 0.2. Together with the optimized polarization configuration with close two-phase content (*R*3*c*, 48.5%; *Pm*-*3m*, 51.5%) at the solid solubility limit of Mn, the BF–BT–0.2Mn exhibited enhanced FOM of PEH, increasing nearly by 2.3 times compared with pure BF–BT (Fig. 6b). Once the Mn content exceeds the solid solution limit, excessive Mn ions enriched in grain boundaries, resulting in grain size reduction and lattice shrinking [41], which will then lead to the decrease of FOM shown in Fig. 6b. Furthermore, before the solid solution limit of Mn addition, some Mn^3+^ ions can act as oxidants to further oxidize Fe^2+^ ions and effectively inhibits the valence change of Fe [42], consequently weaking leakage conductance loss to keep high HT-*ρ*. Of course, when the Mn content exceeds the solid solution limit (*x* = 0.5), this effect is weakened and the *ρ* at 250 °C shows no significant change. Finally, the internal bias field derived from different types of defect dipoles can promote domain growth and maintain domain orientation, enhancing the ability of thermal depolarization resistance (Fig. 6b) before the solid solution limit of Mn addition. As a result, outstanding FOM associated with the power generation of PEH, excellent HT-*ρ* characteristic and high *T*_d_ were simultaneously realized by defect engineering with rationally dopants modulation, in accordance with the previous design strategy forecast, which can be used in HT-PEHs.

### Energy Harvesting Performance

**Fig. 7:**
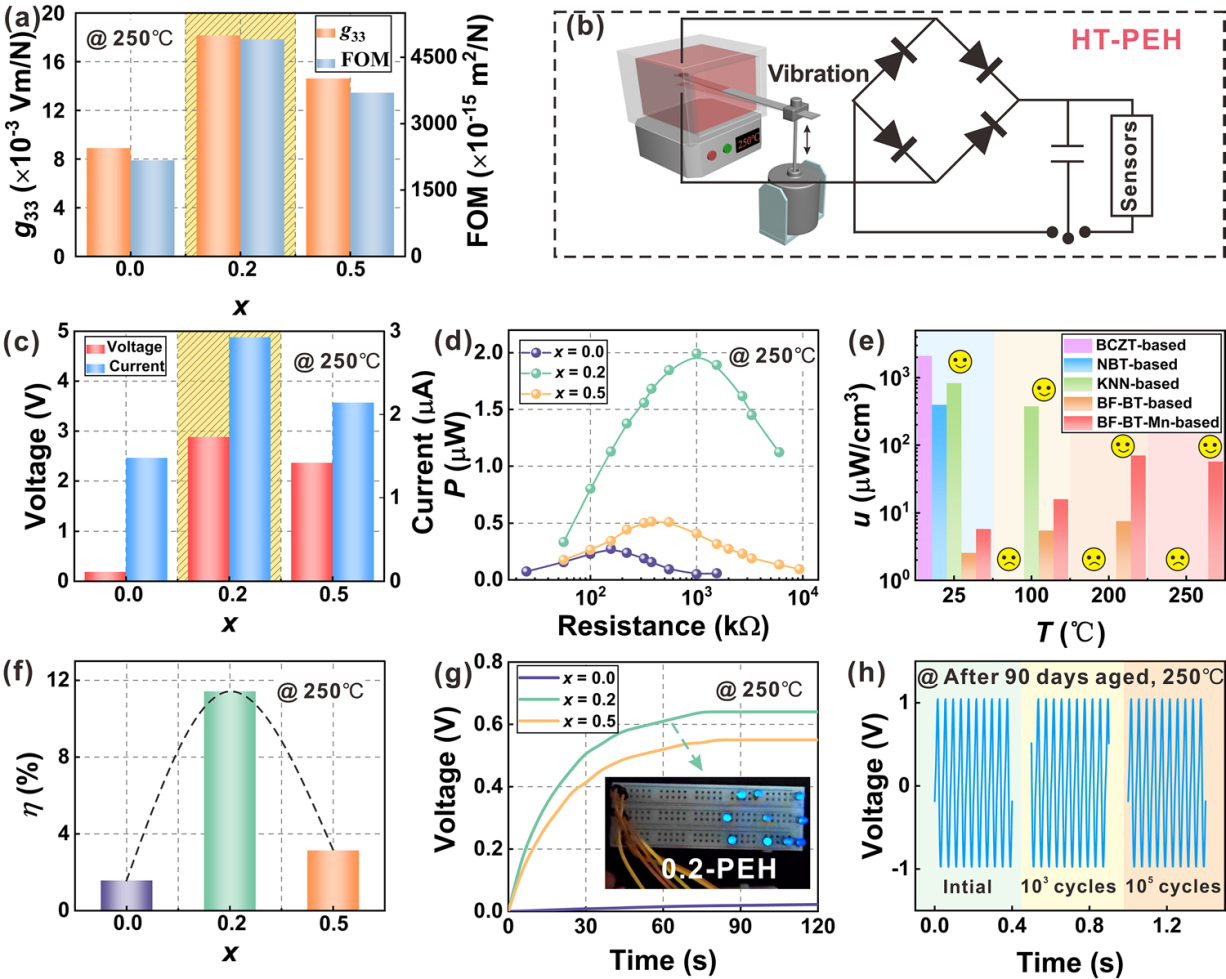
**a** *g*_33_ and FOM of BF-BT-*x*Mn (*x* = 0.0, 0.2, and 0.5) samples measured at 250 °C; **b** Schematic diagram of the PEH test system; **c** *V*_open_ and *I*_SC_ of the three PEHs tested at 250 °C; **d** Output power with load resistance of the three PEHs tested at 250 °C; **e** Comparison of power density for different systems lead-free PEHs at different temperatures; **f** *η* of the three PEHs at 250 °C; **g** Charging curves of the commercial electrolytic capacitor charged by the three PEHs at 250 °C, and the inset shows parallel 10 LEDs lit up; **h** Open circuit voltage of BF–BT–0.2Mn PEH under different vibration cycles at 250 °C after 90 days aged

Figure 7a compares the *g*_33_ and FOM measured at 250 °C for the three samples. Thanks to the defect engineering strategy, the optimal values of the two parameters are achieved simultaneously at the solid solution limit of Mn doping (*x* = 0.2). For BF–BT–0.2Mn ceramic, at 250 °C, the *g*_33_ is 18.2 × 10^–3^ Vm N^−1^ and the FOM is 4905 × 10^–15^ m^2^ N^−1^. Combined with its excellent high-temperature insulation resistance of 10^7^ Ω cm at 250 °C and high *T*_d_, the BF–BT–0.2Mn is the most suitable lead-free piezoceramic for HT-PEH production. In order to evaluate the availability of modified piezoceramics, BF–BT–*x*Mn (*x* = 0, 0.2, 0.5) was assembled into a gradient cantilever beam type HT-PEH for power generation tested at different temperature (25, 100, 200, and 250 °C), 1* g* acceleration and 34 Hz (Fig. 7b), where the test frequency was the resonance frequency of the cantilever beam affixed to piezoceramics (Fig. S9). The results showed that all the three BF-BT-*x*Mn (*x* = 0, 0.2, 0.5) based PEHs can present a stable *V*_open_ and *I*_SC_ signal in the temperature range of 25–250 °C (Fig. S10a-c). Obviously, BF–BT–0.2Mn PEH maintains both high *V*_open_ and large *I*_SC_, mainly due to the fact that it has the largest values of *g*_33_ and *d*_33_ relative to the two counterparts (Fig. 7c). Moreover, Fig. S10d-f shows the output power of three BF–BT-based HT-PEHs at different temperature in an external circuit with different loads. Especially, BF–BT–0.2Mn PEH showed the best power generation capacity at an external load, with an output power of up to 2 μW at 250 °C (Fig. 7d). The power density at 250 °C of BF–BT–0.2Mn PEH (56.96 μW mm^−3^) had a certain attenuation contrast with that at 200 °C (70.23 μW mm^−3^), but compared with other lead-free piezoceramic-based PEH [43–46], the BF–BT–0.2Mn PEH has excellent power generation capacity at 250 °C, which has not been previously reported before, filling the gap in lead-free PEH at high temperatures (Fig. 7e). The energy conversion efficiency (*η*) is also an important parameter to evaluate the ability to convert mechanical energy into electrical energy, which can be calculated by the following formula [47–49]:

9 $$ \eta = \frac{{E_{{{\text{output}}}} }}{{E_{{{\text{input}}}} }} = \frac{P \cdot T}{{\pi \cdot F_{0} \cdot u_{0} }} $$

where *E*_output_ is output electric energy to external resistor, *E*_input_ is input mechanical energy to entire cantilever beam, *P* is the average output power, *T* is an oscillation cycle, *F*_0_ is the amplitude of the harmonic excitation force (*F*_0_ = m*a_0_, *m* is mass of entire cantilever beam, *a*_0_ is amplitude of vibration acceleration), and *u*_0_ is the amplitude of oscillation at position of the centroid. The *η* of the three PEHs at different temperatures is shown in Fig. S11 and the *η* of BF–BT–0.2Mn PEH at 250 °C is as high as 11.43%, increased by about 7 times and 3 times compared with the BF–BT–0Mn PEH and BF–BT–0.5Mn PEH at the same temperature (Fig. 7f), respectively. Thanks to this, BF–BT–0.2Mn PEH shows excellent high temperature fast charging capability for 47 μF commercial electrolytic capacitors (Fig. 7g), while using the stored energy can light up to 10 LEDs in parallel. And after 90 days aged, BF–BT–0.2Mn PEH still maintains excellent output performance without degradation after 10^5^ cycles at 250 °C, showing outstanding vibration fatigue resistance. All the above experiments confirm the feasibility of the defect engineering design strategy in the construction of high temperature piezoceramics, and the optimized Mn doped BF–BT material is expected to be further used in the industrial manufacturing of a new generation of lead-free HT-PEH.

## Conclusions

In summary, a defect engineering strategy with fine-tuned Mn doping is proposed to enhance the high temperature power generation capacity of BF–BT-based piezoceramics. On the one hand, Mn doping can modulate polarization configuration of BF–BT system and optimize the composition of MPB, thus improving the piezoelectric properties; on the other hand, Mn ion can inhibit the valence state fluctuation of Fe ion, thus enhancing the high-temperature insulation characteristics; at the same time, the internal bias field associated with Mn doping can stabilize the domain orientation and increase the depolarization temperature. When the doping amount of Mn ions reaches the solid solution limit (*x* = 0.2), the modified BF-BT ceramics obtain comprehensive excellent electrical properties at 250 °C: the *d*_33_ is 270 pC N^−1^, the *g*_33_ is 18.2 × 10^–3^ Vm N^−1^, the FOM is 4905 × 10^–15^ m^2^ N^−1^ and the *ρ* is maintained at 10^7^ Ω cm orders of magnitude. The cantilever beam-type PEH assembled from BF–BT–0.2Mn piezoceramic shows excellent high-temperature power generation capacity with high *η*, which can realize the rapid charging of commercial electrolytic capacitors and light LED lamp sets, achieving a new breakthrough of lead-free PEH suitable for 250 °C working scenario. The results presented here thus provide guidance to enable defect engineering strategy for the design of lead-free piezoceramics with improved high-temperature energy harvesting performance.

## Supplementary Information

Below is the link to the electronic supplementary material.

Supplementary file1 (DOCX 5670 KB)
