# Supplementary material for: Defect Engineering with Rational Dopants Modulation for High-Temperature Energy Harvesting in Lead-Free Piezoceramics
Source: Nanomicro Lett. 2024 Nov 4;17:55. doi: 10.1007/s40820-024-01556-5 (PMC11534916; doi:10.1007/s40820-024-01556-5)
Supplement: Supplementary file 1 — Supplementary file1 (DOCX 5670 KB) [file 40820_2024_1556_MOESM1_ESM.docx]

Supporting Information for

**Defect Engineering with Rational Dopants Modulation for High-Temperature Energy Harvesting in Lead-Free Piezoceramics**

Kaibiao Xi^1,†^, Jianzhe Guo^1,†^, Mupeng Zheng^1^, Mankang Zhu^1^, Yudong Hou^1,*^

Key Laboratory of Advanced Functional Materials, Ministry of Education, College of Materials Science and Engineering, Beijing University of Technology, Beijing 100124, P. R. China

^†^Kaibiao Xi and Jianzhe Guo contributed equally to this work.

*Corresponding author. E-mail: [ydhou@bjut.edu.cn](mailto:ydhou@bjut.edu.cn) (Yudong Hou)

**Supplementary Figures**


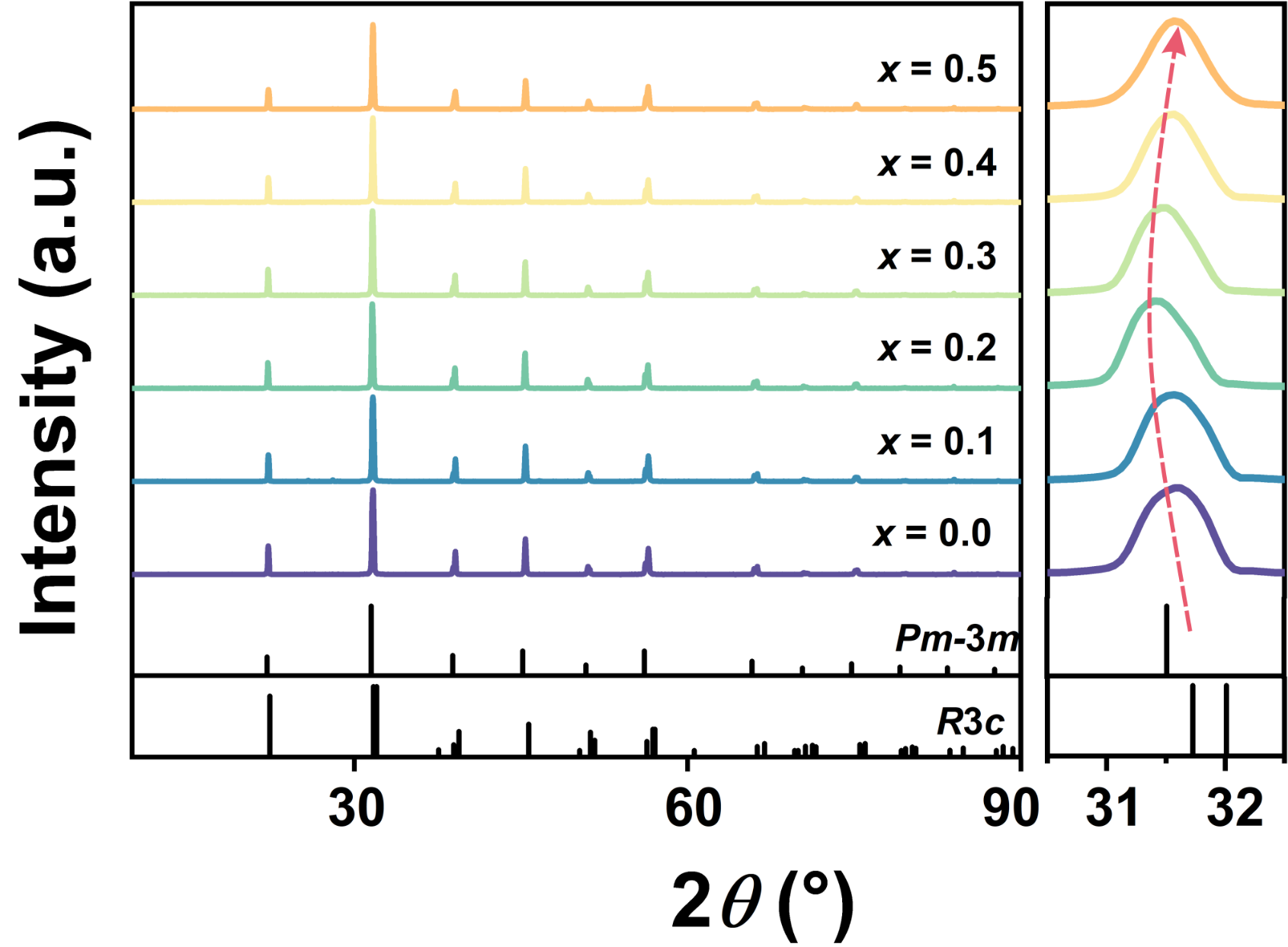


**Fig. S1** The room-temperature XRD patterns of BF-BT-*x*Mn ceramics in the 2*θ* range of 10°-90° and 30.5°-32.5°


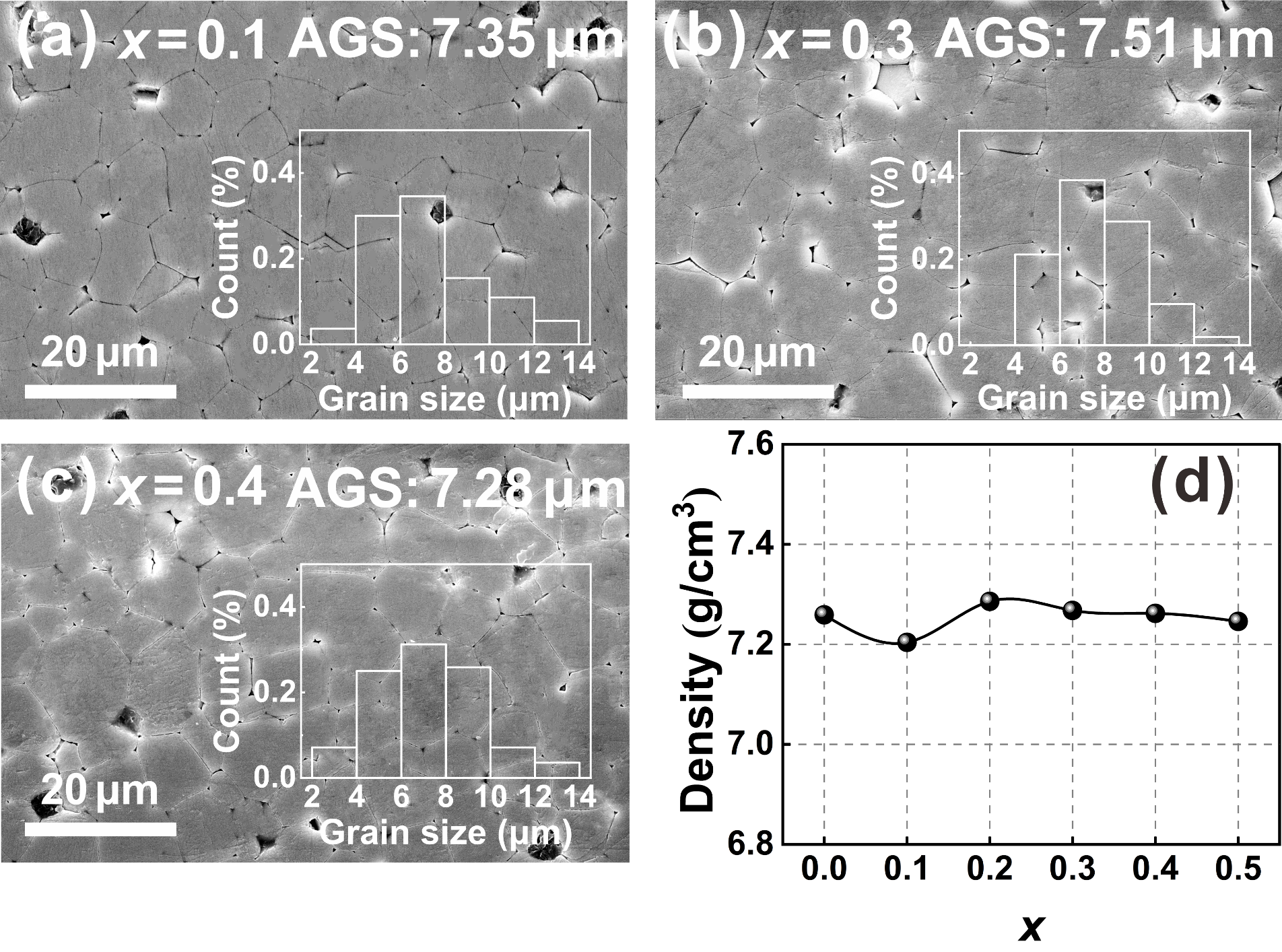


**Fig. S2** (**a-c**) SEM images of the polished and acid etched cross-section of *x* = 0.1, 0.3 and 0.4 samples, and the insets show the corresponding grain size distribution; (**d**) The density of the BF-BT-*x*Mn ceramics


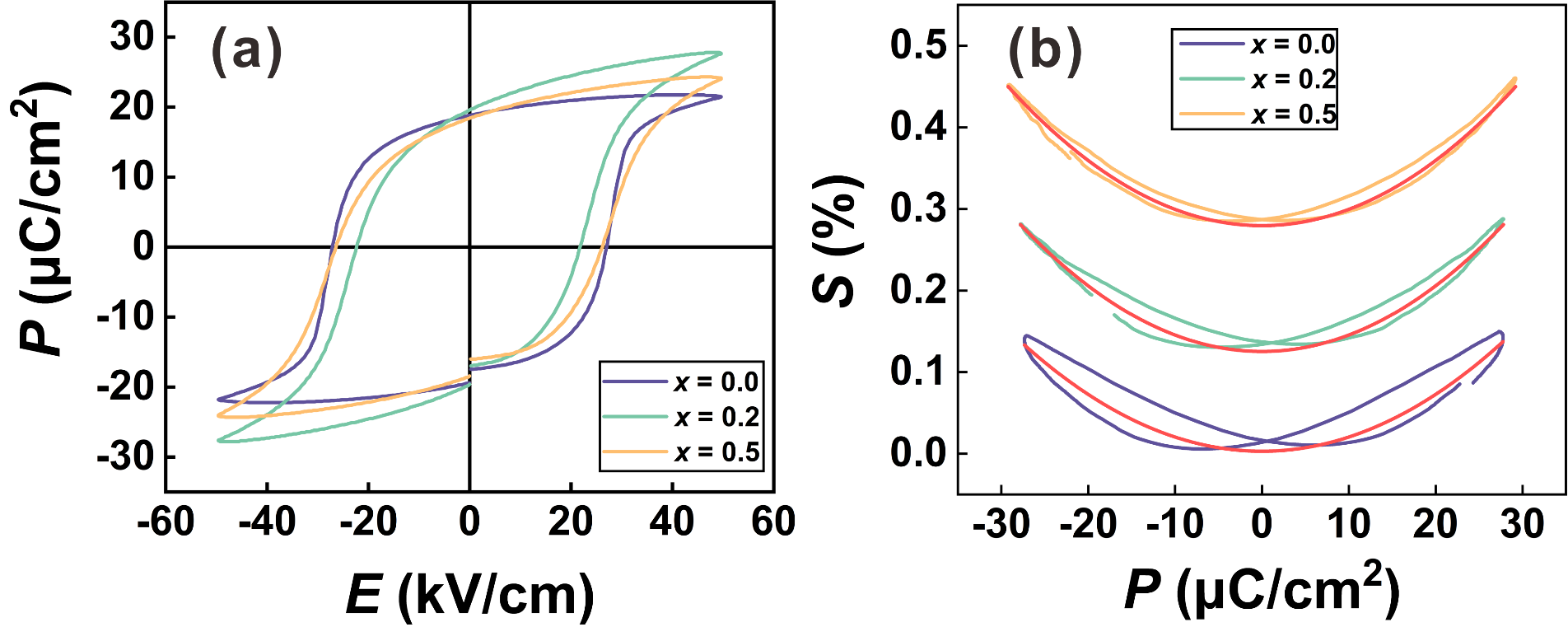


**Fig. S3** (**a**) *P-E* loops and (**b**) *S*-*P* curves of *x* = 0.0, 0.2 and 0.5 samples


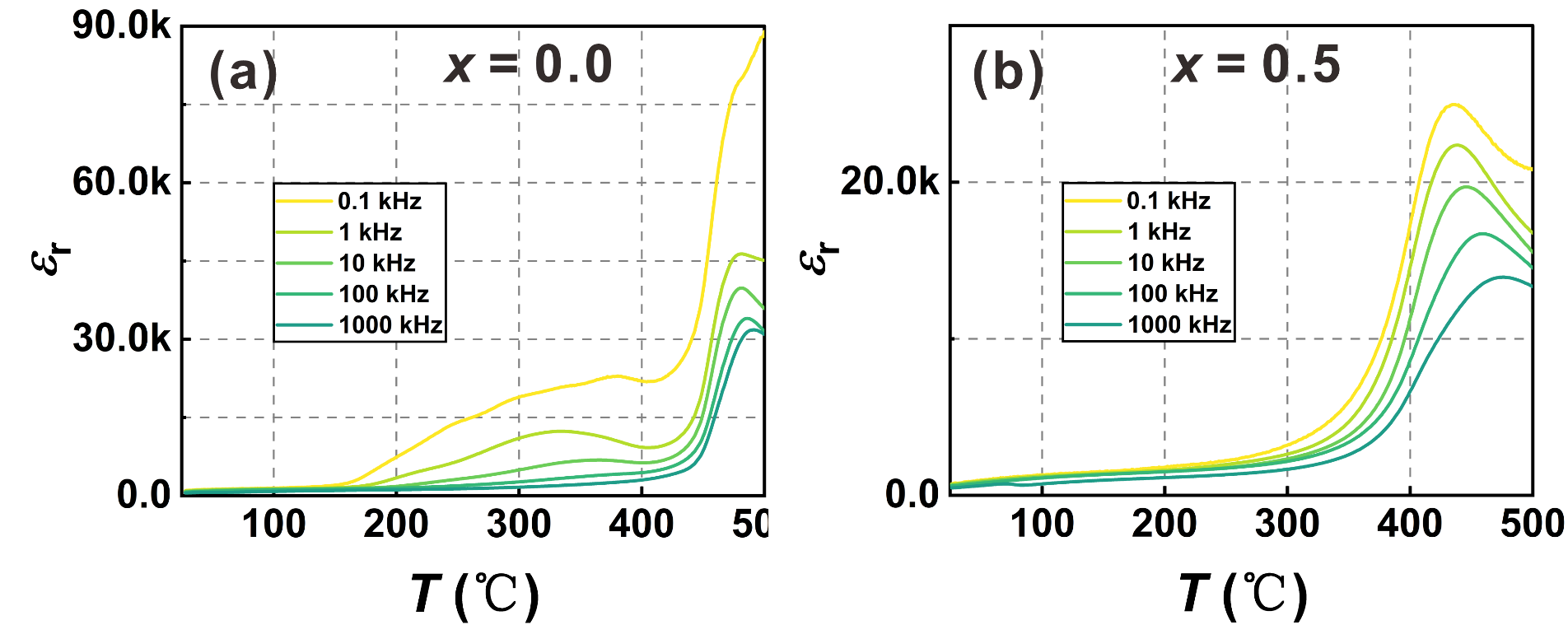


**Fig. S4** The *ε*_r_-*T* curves of (**a**) *x* = 0.0 and (**b**) *x* = 0.5 sample ceramics measured at different frequency


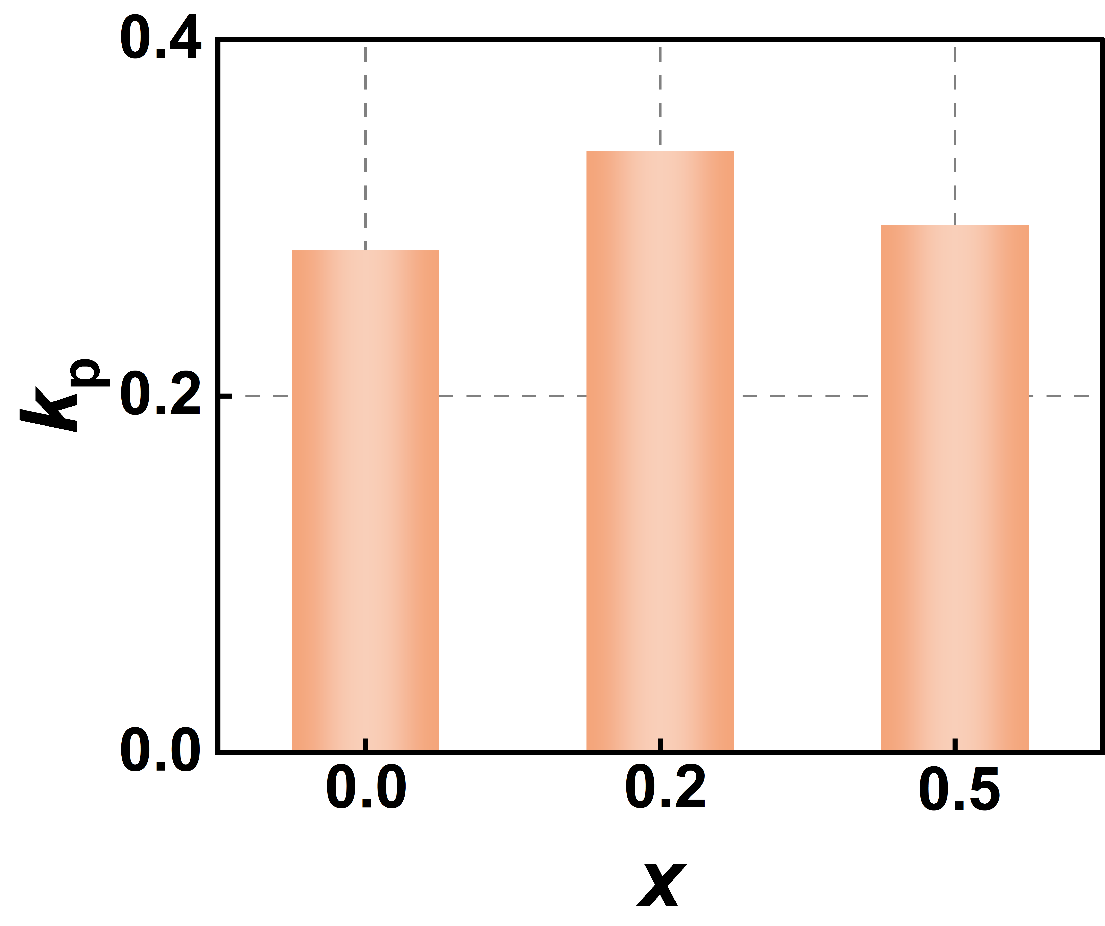


**Fig. S5** The *k*_p_ of the BF-BT-*x*Mn ceramics


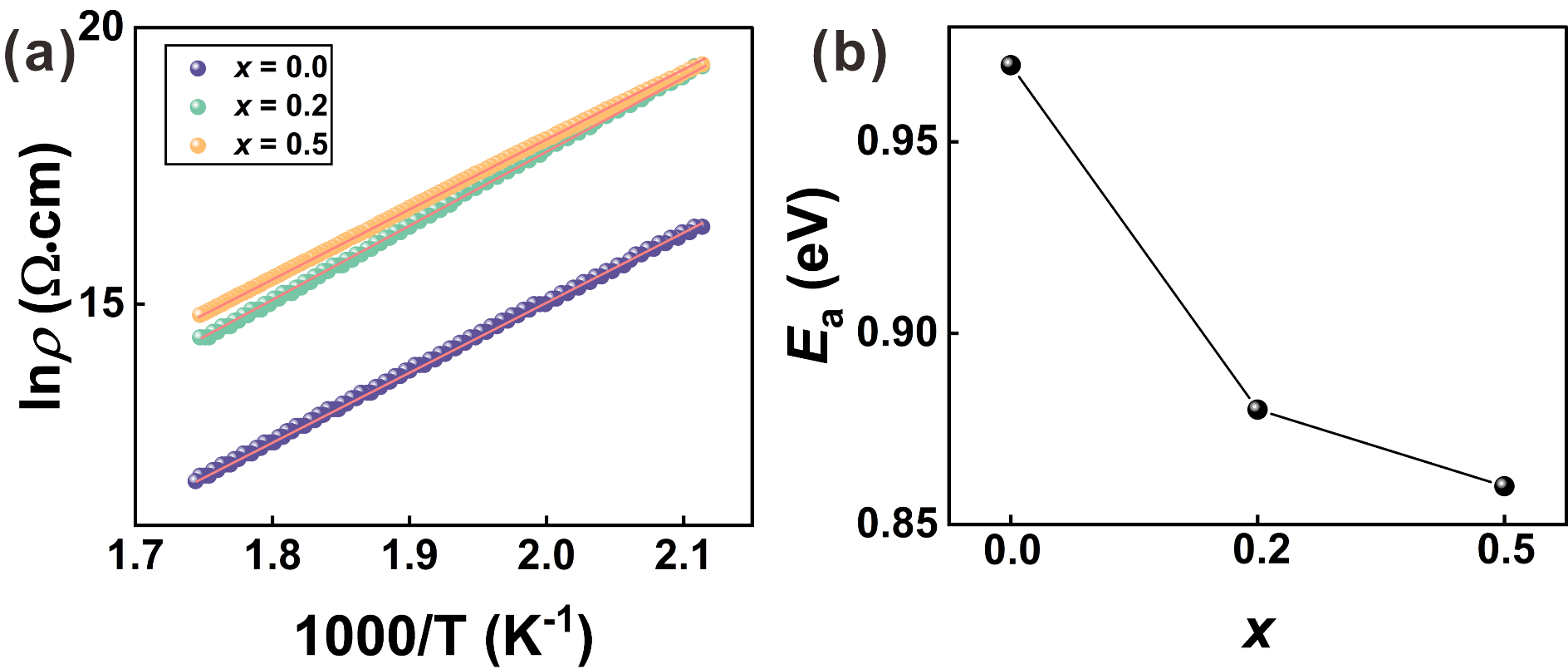


**Fig. S6** (**a**) The ln*ρ* -1000/*T* curves of *x* = 0.0, *x* = 0.2 and *x* = 0.5 ceramics fitted by DC impedance; (**b**) The conductance activation energy *E*_a_ of the three samples


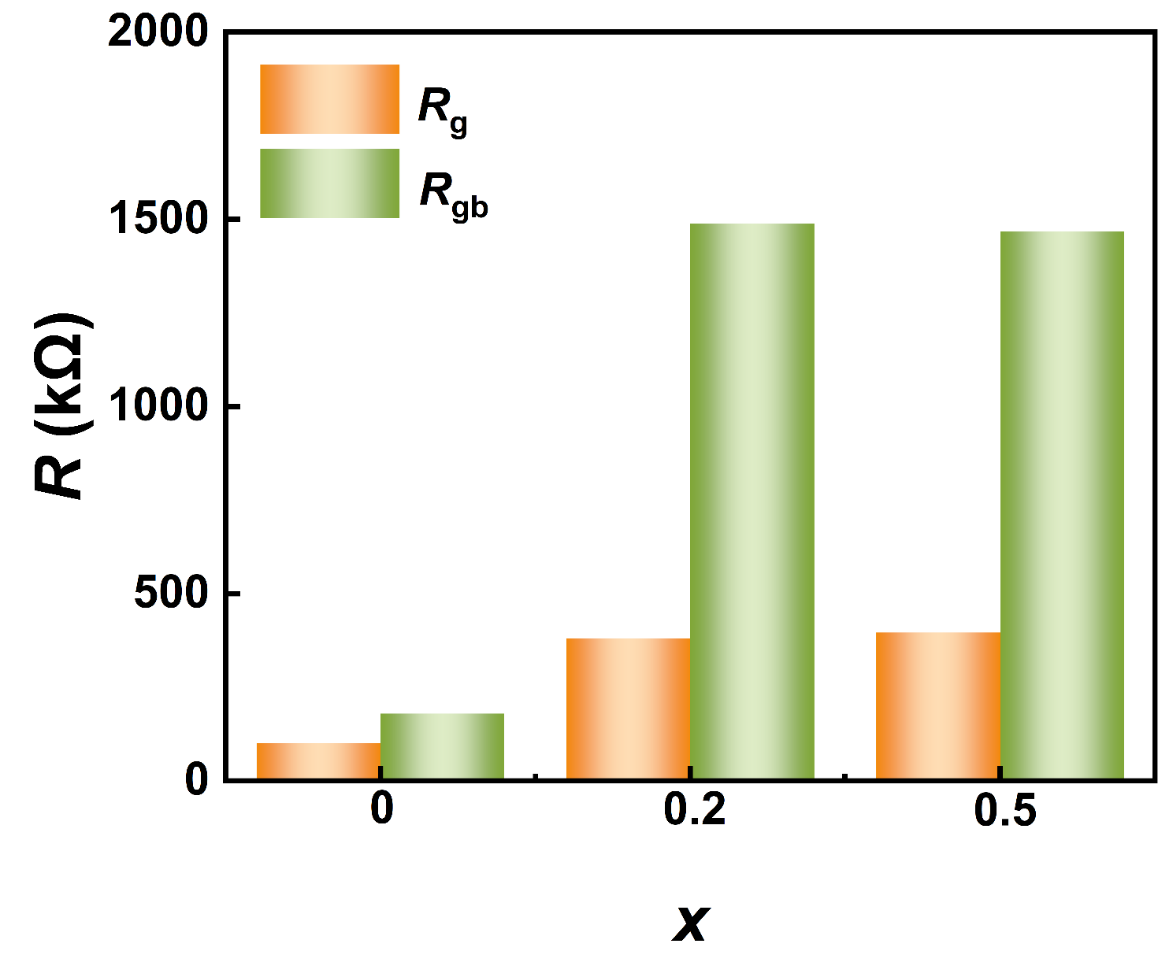


**Fig. S7** *R*_g_ and *R*_gb_ of *x* = 0.0, 0.2 and 0.5 samples measured at 250 ^o^C


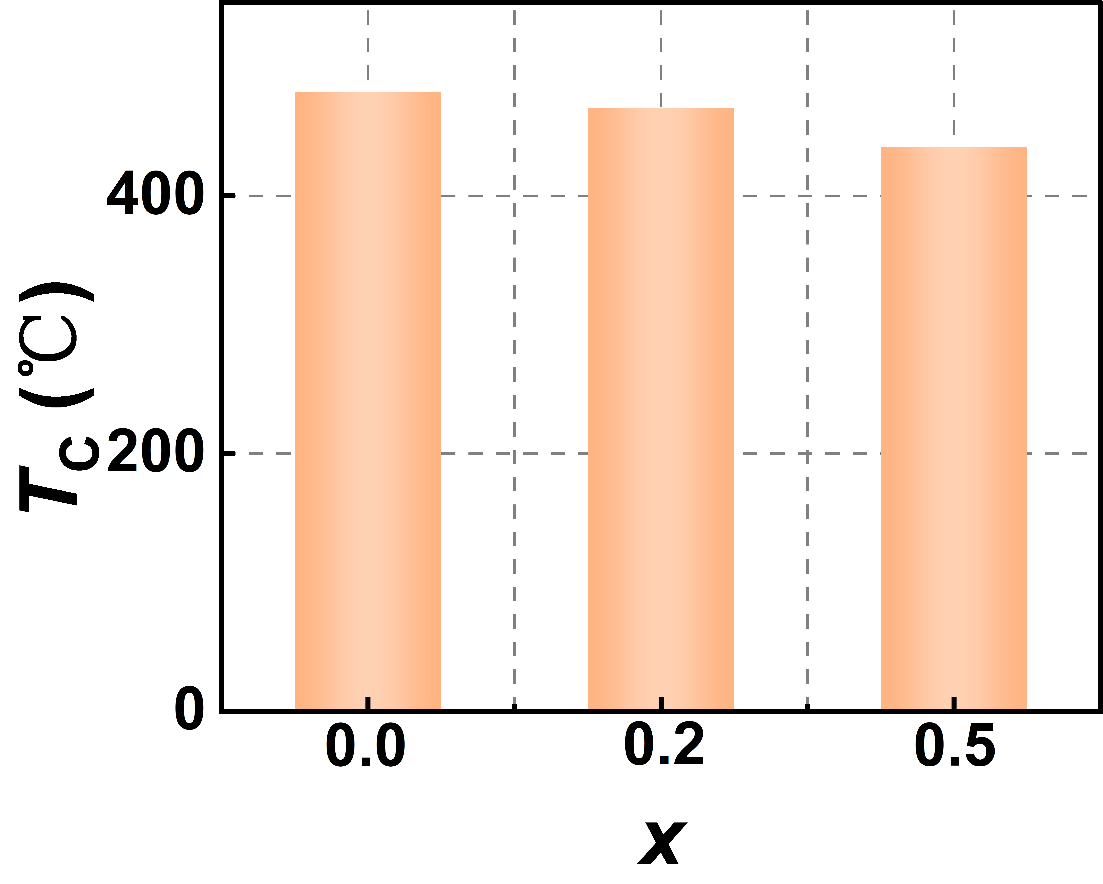


**Fig. S8** The *T*_C_ of the BF-BT-*x*Mn ceramics


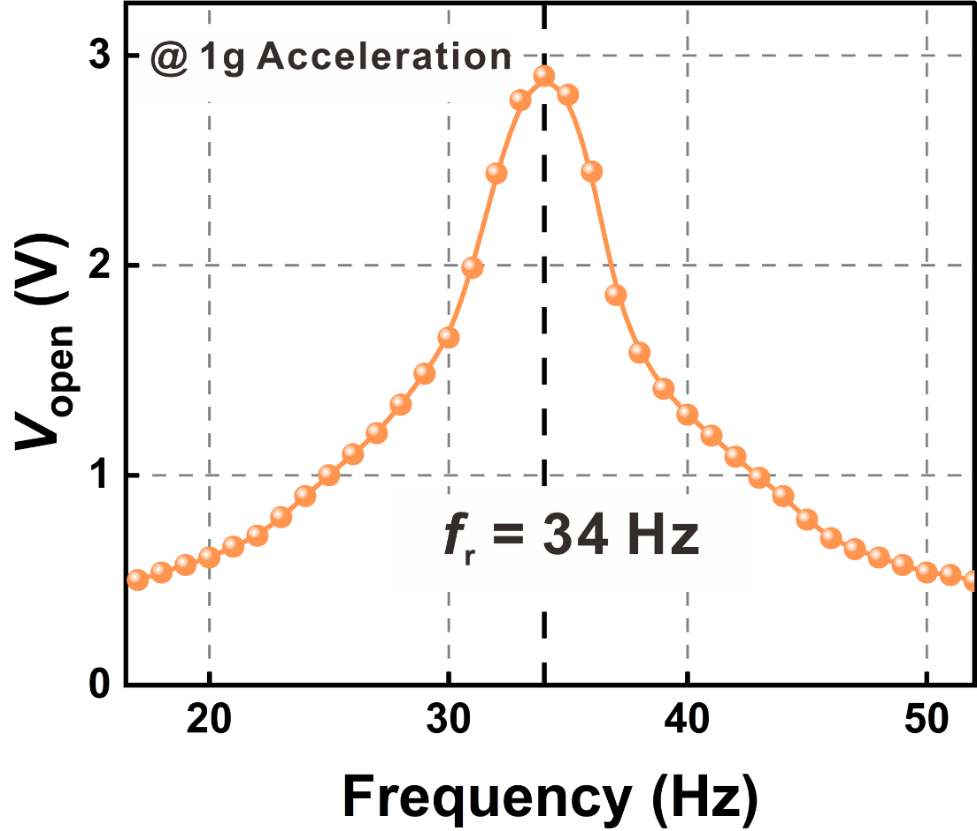


**Fig. S9** The relationship between the *V*_open_ and frequency of the cantilever PEHs system


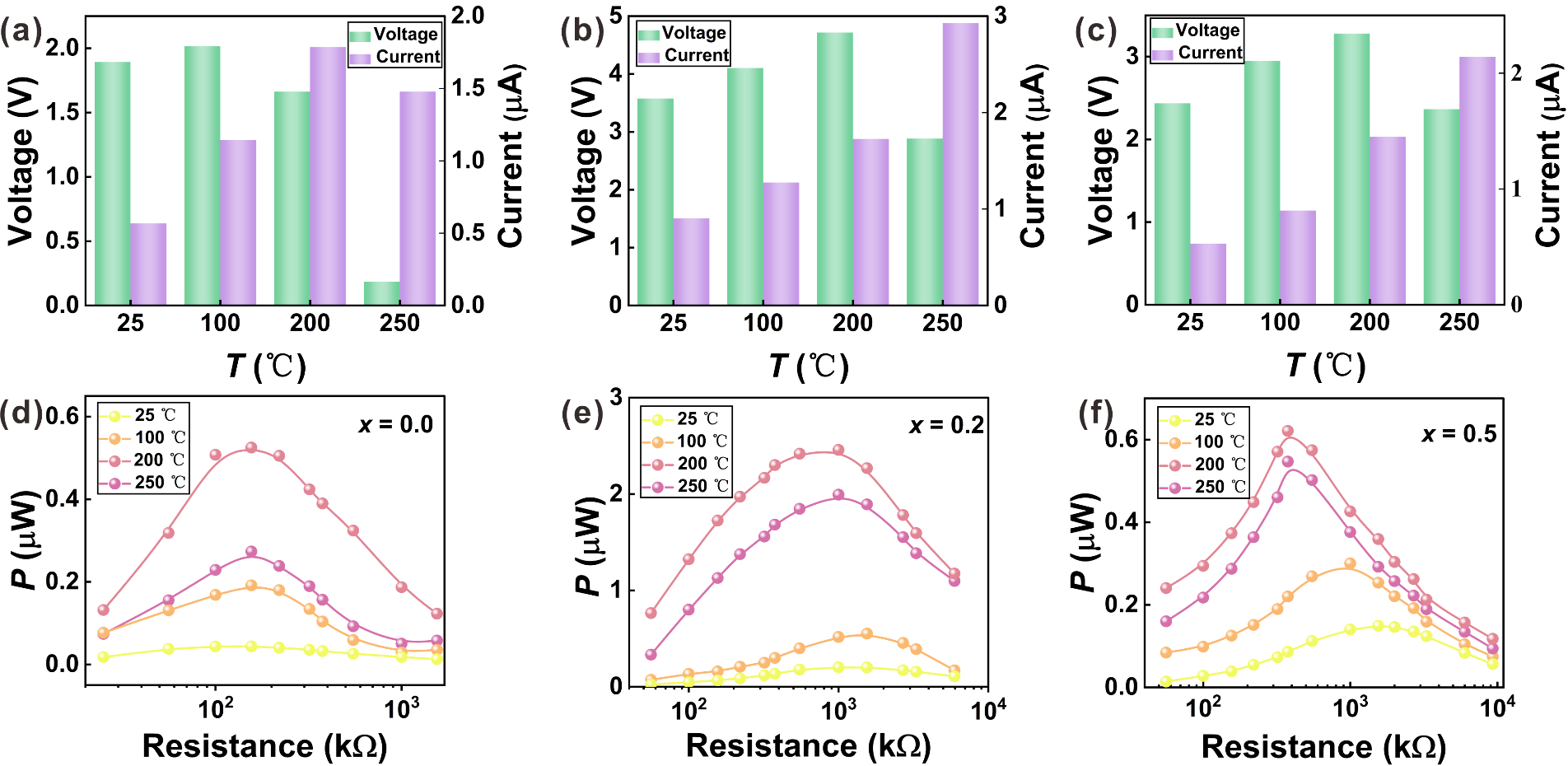


**Fig. S10** (**a-c**) The *V*_open_ and *I*_SC_ of the three PEHs tested at different temperature; (**d-f**) The output power with load resistance of the three PEHs tested at different temperature.


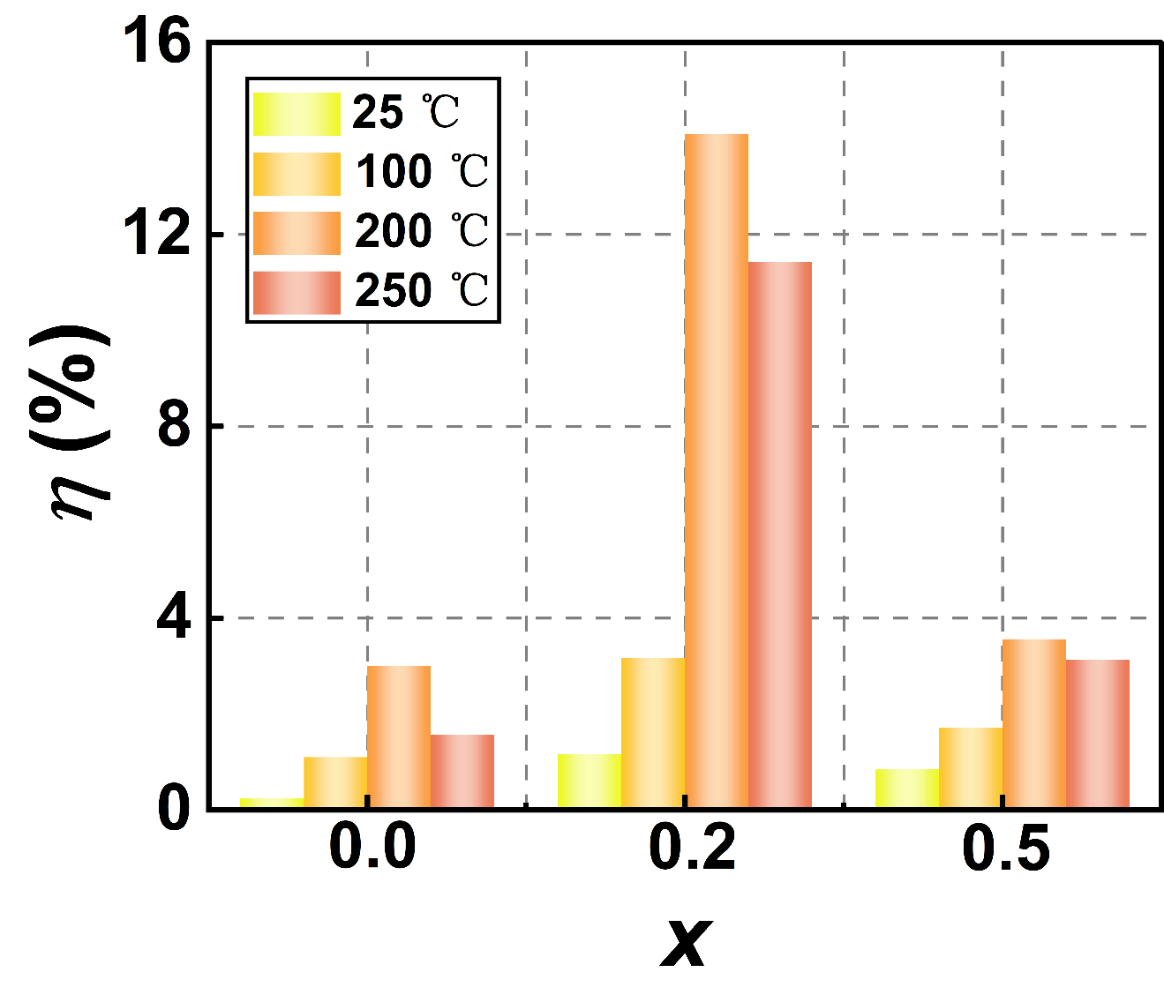


**Fig. S11** The *η* of the three PEHs tested at different temperature

**Table S1** The length of B-O bond of *x* = 0.0, *x* = 0.2 and *x* = 0.5 samples

|  | *R*3*c* (*l*_B-O_ = a/2) | *R*3*c* (*l*_B-O_ = c/2) | *Pm*-3*m* (*l*_B-O_ = c/2) |
| --- | --- | --- | --- |
| *x* = 0.0 | 2.81940 Å | 6.9436 Å | 2.00545 Å |
| *x* = 0.2 | 2.83155 Å | 6.9473 Å | 2.00720 Å |
| *x* = 0.5 | 2.82145 Å | 6.9150 Å | 2.00595 Å |
